# Cancer cachexia induces senescent reprogramming of brown adipose tissue and pro-cachectic S100A9 secretion by adipocytes

**DOI:** 10.1038/s41419-026-08806-x

**Published:** 2026-05-02

**Authors:** Claudia Di Biagio, Flavia Tortolici, Francesco Gaudioso, Andrea Ninni, Francesca Giurdanella Annina, Chiara De Ranieri, Fabio Zaccaria, Francesca Sciarretta, Verteramo Luca, Francesca Arciprete, Simone Carotti, Antoine AF de Vries, Sander Kooijman, Francesca Pacello, Andrea Battistoni, Daniele Lettieri-Barbato, Katia Aquilano

**Affiliations:** 1 Department of Biology, University of Rome Tor Vergata, Rome, Italy; 2 PhD Program in Evolutionary Biology and Ecology, Department of Biology, University of Rome Tor Vergata, Rome, Italy; 3 PhD Program in Ecology, Evolution and Environmental Health, Department of Biology, University of Rome Tor Vergata, Rome, Italy; 4 Microscopic and Ultrastructural Anatomy Research Unit, Department of Medicine and Surgery, Università Campus Bio-Medico di Roma, Rome, Italy; 5 Department of Cardiology, Laboratory of Experimental Cardiology, Leiden University Medical Center, Leiden, The Netherlands; 6 Department of Medicine, Division of Endocrinology, and Einthoven Laboratory for Experimental Vascular Medicine, Leiden University Medical Center, Leiden, The Netherlands; 7 IRCCS Fondazione Bietti, Rome, Italy

**Keywords:** Mechanisms of disease, Cancer models

## Abstract

Cancer-associated cachexia (CAC) is a multifactorial wasting syndrome characterized by progressive loss of fat and lean mass, systemic inflammation, and poor therapeutic responsiveness. While brown adipose tissue (BAT) is traditionally considered a protective, energy-dissipating organ, its qualitative remodeling in CAC remains poorly characterized.

Here, we demonstrate that CAC induces a senescent conversion of BAT, marked by thermogenic failure, fibrosis, inflammation, and acquisition of a senescence-associated secretory phenotype (SASP). Through integrative transcriptomic, proteomic, and secretomic analyses in a murine model of lung cancer-induced cachexia, we identify S100A9 as a key factor selectively upregulated and secreted by brown adipocytes. Functional assays reveal that the BAT secretome exerts deleterious paracrine effects on white adipocytes and skeletal myotubes, promoting lipolysis and atrophy, while also impairing brown adipocyte identity in an autocrine manner. Co-culture and gain-of-function experiments with S100A9 recapitulate these phenotypes in vitro in mouse and human brown adipocytes, whereas pharmacological blockade of S100A9 signaling partially restores thermogenic and metabolic features. Collectively, our findings reveal that BAT undergoes functional reprogramming into a senescent and secretory tissue in cancer cachexia, with adipocyte-derived S100A9 acting as a novel pro-cachectic mediator. This work redefines the role of BAT in CAC and identifies S100A9 as a potential therapeutic target within the adipose–muscle crosstalk.

## Introduction

Cancer-associated cachexia (CAC) is a multifactorial metabolic syndrome characterized by unwanted weight decrease, progressive loss of lean and fat mass, and systemic inflammation [1–3]. Depending on the cancer type, approximately 50% to 80% of patients develop CAC [1, 4], characterized by severe physical impairment and reduced therapeutic efficacy, ultimately contributing to up to 30% of cancer-related deaths [5, 6]. The underlying mechanisms of CAC imply intricate crosstalk between tumor and host tissues, impinging coordinated catabolic remodeling of both muscles and adipose depots [2, 3]. In skeletal muscle, proteolysis is prominently activated through the ubiquitin–proteasome pathway and the autophagy–lysosome system, both of which converge to accelerate the degradation of contractile proteins and organelles. Muscle-specific E3 ubiquitin ligases such as MuRF1 and Atrogin1 are upregulated and trigger transcriptional programs promoting atrophy [7–9].

Adipose tissue is increasingly recognized as a dynamic endocrine organ actively participating in the progression of CAC. In white adipose tissue (WAT), profound metabolic and histomorphological rearrangements are observed, characterized by inflammation and elevation of lipolytic processes via upregulation of lipases [10, 11]. Moreover, transition towards a brown-like phenotype, increasing thermogenic UCP1 protein, is observed with consequent enhanced energy expenditure [12–14]. These alterations precede overt weight loss, implicating dysfunctional adipose signaling as an early and active driver of CAC [11, 14].

In contrast, the role of brown adipose tissue (BAT) in CAC remains poorly understood. While BAT is specialized for thermogenesis and lipid oxidation, evidence from pre-clinical models and selected patient cohorts suggests it may contribute to wasting exacerbation via increased energy dissipation [14, 15]. Though other human data show inconsistent associations between BAT activity, cachexia severity, and increased mortality [16–18].

These catabolic cascades are driven by tumor- and host-derived mediators, including inflammatory cytokines and cachexigenic factors (e.g., IL-1β, IL-6, PTHrP), contributing to the systemic hypercatabolic milieu [12, 19–21]. Among emerging mediators, GDF-15 has gained prominence for its ability to promote CAC and systemic wasting by suppressing appetite and directly altering lipid handling and mitochondrial function in WAT and skeletal muscle, contributing to fat and lean mass loss [22–24]. Yet, these factors do not fully explain the heterogeneity and persistence of cachexia, prompting investigation into additional catabolic drivers.

In this context, S100A9 has emerged as a novel inflammatory alarmin implicated in muscle and adipose wasting and CAC [25–27]. S100A9 is a calcium-binding protein that can form heterodimers with S100A8 to form calprotectin and acts via the engagement of RAGE and TLR4 membrane receptors to amplify inflammatory responses. S100A9 also exerts biological activity independently. Indeed, several studies have shown that S100A9 forms stable homodimers that can bind TLR4 or RAGE and trigger pro-inflammatory signaling, leading to cytokine production and catabolic responses, also in situations of tumor burden [28, 29]. S100A9 is typically produced by neutrophils and monocytes, and while its transient expression aids in tissue repair, chronic elevation promotes persistent inflammation and tissue degeneration. In cancer models, circulating S100A9 levels correlate with body weight loss, and S100A9 directly induces muscle atrophy in vitro [25].

Importantly, both WAT and BAT secrete a diverse range of bioactive molecules able to modulate systemic metabolism [30]. In cachexia, the secretory profile of WAT shifts toward a pro-inflammatory phenotype, with reduced leptin and elevated adiponectin levels, and increased expression of IL-6, TNF-α, and IL-1β [31]. While BAT is increasingly recognized as an endocrine organ, its exact contribution to systemic wasting in cancer cachexia—and the identity of key batokines mediating crosstalk with muscle and WAT—remains incompletely characterized.

Despite the growing interest in S100A9 as a cachexigenic factor, its contribution from adipose tissues—and particularly from BAT—has not been investigated. Whether BAT acquires a pathologic phenotype that includes S100A9 secretion during CAC is unknown.

Here, we address this gap using a murine model of lung cancer-associated cachexia. We show that BAT undergoes profound remodeling marked by suppression of thermogenic programs, fibrosis, and acquisition of a senescence-associated secretory phenotype (SASP). We identify brown adipocytes as a novel source of S100A9 and demonstrate its role in driving cachexia features. These findings reposition BAT as an active contributor to systemic wasting and uncover S100A9 as a potential therapeutic target in CAC.

## Materials and methods

### Cell cultures

All cell lines were maintained at 37 °C in a humidified atmosphere with 5% CO₂ and used at passages ≤ 8. Cells were routinely tested for Mycoplasma contamination using the N-GARDE Mycoplasma PCR Reagent Set (Euroclone), according to the manufacturer’s instructions, and were confirmed to be Mycoplasma-negative before use.

### Murine brown adipocytes

The conditionally immortalized murine brown preadipocyte cell line (mBA) was kindly provided by Prof. Sander Kooijman (Dept. of Medicine, Division of Endocrinology, and Einthoven Laboratory for Experimental Vascular Medicine, Leiden University Medical Center, The Netherlands) and Prof. A.A.F Antoine de Vries (Dept of Cardiology, Laboratory of Experimental Cardiology, Leiden University Medical Center, The Netherlands). Cells were cultured in DMEM/F12 medium (Euroclone) supplemented with 25 mM HEPES (Gibco), 365 mg/L L-glutamine (Euroclone), 10% heat-inactivated newborn calf serum (NCS) (ATCC), 1% Penicillin/Streptomycin (Euroclone), and 0.1 μg/mL doxycycline (Sigma-Aldrich). For differentiation into mature adipocytes, cells were seeded at a density of 8000 cells/cm² in the presence of doxycycline on plates pre-coated with 0.2% gelatine in sterile bidistilled H₂O (ddH₂O). After 72 h, at approximately 80–90% confluence, the medium was replaced with differentiation medium consisting of DMEM/F12 supplemented with 0.1 mg/mL insulin (Sigma-Aldrich), 10 mM HEPES, 10 μM rosiglitazone (Sigma-Aldrich), and 25 ng/mL ascorbic acid (Gibco). The differentiation medium was refreshed every 48 h until differentiation into mature brown adipocytes, achieved after 8 days.

The murine brown preadipocyte cell line T37i was kindly provided by Prof. Marc Lombès (INSERM U478, Faculty of Medicine, Paris, France). Cells were cultured in DMEM/F12 medium containing 25 mM HEPES, 365 mg/L L-glutamine, 10% heat-inactivated FBS (Euroclone), and 1% Penicillin/Streptomycin. Preadipocytes were seeded at a density of 2500 cells/cm². After 72 h, when cells reached approximately 70–80% confluence, the medium was replaced with differentiation medium consisting of the growth medium supplemented with 0.1 mg/mL insulin, 10 μM rosiglitazone, and 0.2 μM triiodothyronine T3 (Sigma-Aldrich). The medium was refreshed every 48 h until full differentiation, 8 days post-induction.

### Human brown adipocytes

The conditionally-immortalized human brown preadipocyte cell line (hBA) was kindly provided by Prof. Sander Kooijman and Prof. A.A.F Antoine de Vries (Leiden University Medical Center, Dept. of Medicine, Leiden, The Netherlands). Cells were cultured in complete DMEM/F12 medium containing 25 mM HEPES, 365 mg/L L-glutamine, 10% heat-inactivated FBS, 1% Penicillin/Streptomycin, and 0.1 μg/mL doxycycline. For differentiation, cells were seeded at a density of 30,000 cells/cm² in the presence of doxycycline on plates pre-coated with 0.2% gelatin in ddH₂O. After 72 h, at approximately 80–90% confluence, the medium was replaced with induction medium consisting of DMEM/F12 supplemented with 0.1 μg/mL insulin, 2 nM T3, 1 μM dexamethasone (Sigma-Alrich), 0.5 mM IBMX (Sigma-Aldrich), 1 μM rosiglitazone, and 125 μM indomethacin (Sigma-Aldrich). The induction medium was maintained for 4 days, followed by medium changes every 48 h using differentiation medium consisting of DMEM/F12 supplemented with 0.1 μg/mL insulin, 1 μM rosiglitazone, and 1 nM T3 until complete differentiation into mature brown adipocytes, achieved after 16 days.

### Murine white adipocytes

The 3T3-L1 preadipocyte cell line obtained from ATCC (#CL-173) was cultured in DMEM High Glucose, HG (Thermo Fisher Scientific) containing 4.5 g/L D-glucose, 0.11 g/L sodium pyruvate, 10% heat-inactivated NCS, 1% Penicillin/Streptomycin, and 2 mM L-glutamine. To induce differentiation, preadipocytes were seeded at a final density of 10,000 cells/cm². After 72 hours, the growth medium was replaced with differentiation medium consisting of complete DMEM HG medium supplemented with the following hormones: 1 μg/mL insulin, 1 μM dexamethasone, and 0.5 mM IBMX. After 48 h, the medium was replaced with maintenance medium consisting of complete DMEM HG medium supplemented with 1 μg/mL insulin for 5 days. The medium was refreshed every 48 h until full differentiation into mature white adipocytes, which occurred 8 days after induction.

### Lung cancer cells

The murine Lewis Lung Carcinoma (LLC) and the human A549 lung cancer cell lines were obtained from ATCC (#CRL-1642 and #CCL-1855, respectively). Cancer cells were cultured in DMEM HG supplemented with 10% heat-inactivated FBS, 1% Penicillin/Streptomycin, and 2 mM L-glutamine. Cells were plated the day before the experiment at a density of 15,000 cells/cm².

### Murine macrophages

The murine macrophage cell line RAW264.7 was purchased from ATCC (#TIB-71) and cultured in DMEM HG supplemented with 0.11 g/L sodium pyruvate, 10% heat-inactivated FBS, 1% Penicillin/Streptomycin, and 2 mM L-glutamine. Cells were plated the day before the experiment at a density of 15,000 cells/cm².

### Murine myoblasts

C2C12 myoblasts were obtained from ATCC (#CRL-1772), maintained in DMEM HG supplemented with 10% heat-inactivated FBS, 1% Penicillin/Streptomycin, and 2 mM L-glutamine. For differentiation, cells were seeded and upon reaching confluence, growth medium was replaced with differentiation medium, composed of DMEM HG supplemented with 2% horse serum. Cells were allowed to differentiate for 5–7 days.

### In vitro treatments

#### Adipocytes-lung cancer cells co-culture

Following differentiation, brown adipocytes were co-cultured with LLC or A549 cells. LLC or A549 cells were seeded at a density of 20,000 cells/cm² in cell culture inserts (0.4 μm pore size) and allowed to sediment for 30 min at 37 °C/5% CO₂. The inserts were consequently transferred onto well plates containing the mature adipocytes. Control adipocytes were cultured without inserts. Co-cultures were maintained for 24 h. To specifically assess the secretion of S100A9 by brown adipocytes in response to tumor-derived soluble factors, the inserts containing cancer cells were removed after the co-culture period, and the co-culture medium was completely discarded. Brown adipocytes were then extensively washed and incubated in fresh culture medium for an additional 24 h. Conditioned medium collected during this second incubation period (i.e., in the absence of cancer cells) was used for downstream analyses and is hereafter referred to as the *recovery* condition.

#### Adipocytes-macrophages co-culture

Following differentiation, brown adipocytes were co-cultured with RAW264.7 murine macrophages. RAW264.7 cells were previously seeded at a density of 20,000 cells/cm² in cell culture inserts (0.4 μm pore size). Macrophages were stimulated with 500 ng/mL *E. coli* lipopolysaccharide (LPS) for 6 h. After treatment, the LPS-containing medium was discarded, cells were washed three times with PBS, fresh medium was added, and the inserts were subsequently transferred onto plates seeded with mature adipocytes. Control adipocytes were cultured without inserts. Co-culture was maintained for 24 or 48 h, depending on the experimental design. To specifically assess the secretion of S100A9 by brown adipocytes in response to the interaction with macrophages, the *recovery* medium was employed (see Adipocytes–lung cancer cells co-culture).

#### S100A9 overexpression and treatments

Following differentiation, T37i brown adipocytes were transfected with 250 ng/mL of a plasmid encoding S100A9 (S100A9+) (#MG225450, OriGene) or with an empty vector (Empty) as a control, using Lipofectamine™ 2000 (Thermo Fisher Scientific), according to the manufacturer’s instructions.

To functionally block S100A9 signaling, the S100A9 modulator Tasquinimod (TQ) (#HY-10528/CS-0898, MedChemExpress) was added to the culture medium 8 h post-transfection—after Lipofectamine removal and medium refreshment—and maintained for 24 h.

#### Recombinant S100A9 protein expression, purification, and treatment

Differentiated 3T3-L1, T37i adipocytes, or C2C12 myotubes were treated with recombinant mouse S100A9 (rS100A9) protein purified from *E. coli*. Starting from a stock concentration of 300 μg/mL, the protein was serially diluted to obtain final concentrations of 0, 10, 100, and 1000 ng/mL to identify the biologically effective dose. Hence, a concentration of 1000 ng/mL was used for all the following experiments. Alternatively, commercially available rS100A9 (#2065-59, Novus Biologicals) was also used at same concentrations.

The S100A9 protein was expressed and purified as follows. A synthetic gene encoding mouse S100A9, codon‑optimized for *E. coli*, was synthesized by GeneScript Biotech and cloned into the *Nde*I and *Xho*I restriction sites of pET41a, yielding the plasmid pET41a‑MS100A9. For protein overproduction, the expression plasmid was transformed into *E. coli* BL21(DE3) cells. Overnight cultures grown in LB medium supplemented with 50 µg/mL kanamycin were diluted 1:100 into fresh LB medium containing the same antibiotic and incubated at 37 °C with shaking at 150 rpm. Protein expression was induced with 500 µM IPTG at an OD₆₀₀ of ~0.6, followed by incubation at 37 °C for 3.5 h. Cells were harvested by centrifugation, yielding approximately 2–3 g of wet cell mass per liter of culture, and resuspended in 50 mM Tris-HCl, 100 mM NaCl, 10 mM β-mercaptoethanol (BME), 1.0 mM EDTA, 0.5% Triton X-100, and 1 mM PMSF, pH 8.0. Cells were sonicated on ice (30 s on, 10 s off for 2.5 min; 40% amplitude). The crude lysate was clarified by centrifugation (16,000 × *g*, 35 min, 4 °C), and the supernatant was decanted. The pellet was resuspended in the same buffer, sonicated, and centrifuged again as described above. The resulting cell pellet, containing most of the rS100A9 protein, was resuspended in 50 mM Tris-HCl, 100 mM NaCl, 10 mM BME, and 4 M guanidine hydrochloride (Gdn-HCl), pH 8.0, by gentle stirring at 4 °C. The suspension was incubated overnight at 4 °C, then sonicated (30 s on, 10 s off for 5 min; 40% amplitude) on ice and centrifuged (22,000 × *g*, 35 min, 4 °C). The solubilized proteins were extensively dialyzed against 20 mM HEPES, 10 mM BME, pH 8.0. Prior to loading onto a Q-Sepharose FPLC column, the proteins were centrifuged (16,000 × *g*, 35 min, 4 °C). The column was equilibrated in 20 mM HEPES, 10 mM BME, pH 8.0, and proteins were eluted using a 0–1 M NaCl gradient. Fractions containing S100A9 were pooled, concentrated by ultrafiltration using an Amicon apparatus with a 3 kDa cut-off membrane, and loaded onto a Superdex 75 16/60 column equilibrated with 20 mM HEPES, 100 mM NaCl, and 10 mM BME, pH 8.0. S100A9-containing fractions were pooled, dialyzed against 20 mM HEPES and 10 mM BME, pH 7.0, loaded onto a Q-Sepharose, and eluted using a 0–1 M NaCl gradient. To remove residual contaminants, S100A9 fractions were pooled, dialyzed against 20 mM HEPES, 10 mM BME, pH 6.0, concentrated using an Amicon apparatus, and loaded onto an SP Sepharose Fast Flow cation-exchange column. Fractions containing pure rS100A9 were collected, concentrated, and dialyzed against 16.6 mM Tris-HCl and 0.83 mM Tris(2-carboxyethyl) phosphine (TCEP), pH 8.2, for subsequent use in cell culture experiments.

#### Tasquinimod treatment

For experiments involving TQ treatment, rS100A9 protein was pre-incubated with 20 μM TQ for 1 h at 37 °C in culture medium. This mixture was then added to differentiated T37i adipocytes. Cells were subsequently incubated with the rS100A9 + TQ mixture for 24 h.

#### Mice

Mouse experimentation was conducted in accordance with accepted standard of humane animal care after the approval by relevant local (The University Animal Welfare Committee-OPBA, Tor Vergata University) and national (Ministry of Health, Legislative Decree No. 26/2014; European Directive 2010/63/UE) committees with authorization n° 27/2021-PR. Mice were maintained at 21.0 ± 0.5 °C under a 12 h/12 h light/dark cycle (lights on at 6:00 a.m., lights off at 6:00 p.m.). Food and water were provided *ad libitum*. Mice were maintained on a standard rodent chow diet (3.1 kcal/g, ~18.6% protein and ~6.2% fat, Envigo Teklad Global), and daily food intake was recorded.

For the cancer cachexia model, 8–10-week-old male or female C57BL/6J mice from The Jackson Laboratory (strain no. 000664) were randomly divided into two groups. One group was subcutaneously injected in the right flank with 3 × 10⁶ LLC cells suspended in 100 μl sterile PBS. Control mice were injected with vehicle PBS alone (CTR). Following tumor inoculation, mice were monitored for 21 days to assess changes in tumor size and body weight, hence a cancer-associated cachexia (CAC) phenotype. Only mice that developed tumor mass were included in the study. CTR and CAC mice were weighted, and subcutaneous tumor mass was measured in vivo using a Vernier caliper. To estimate tumor volume, the two main dimensions (length and width) were measured. The percentage of body weight loss was used to calculate the cachexia index, an indicator of the severity of cachexia developed in tumor-bearing mice. The cachectic index was determined by estimating tumor mass based on the assumption that 1 cm³ corresponds to 1 g. This estimated tumor weight was subtracted from the total body weight of the animal to obtain the net body weight. The net weight was then expressed as a percentage of the initial body weight to calculate the percentage of weight loss, defined as the cachectic index.

#### BAT secretome

To analyze the soluble factors released by brown adipose tissue, BAT from CTR or CAC mice was explanted and cultured ex vivo at 37 °C in a 5% CO₂ atmosphere, as previously described [32]. After 24 h, the culture medium was passed through a 70 μm cell strainer and centrifuged to remove cellular debris. The resulting supernatant was used as the BAT-derived secretome.

#### Ex vivo rS100A9 injection in BAT

BAT was aseptically explanted and immediately placed in high-glucose DMEM (4.5 g/L glucose). Recombinant mouse S100A9 (rS100A9; #2065-59, Novus Biologicals) was diluted in PBS at 3 μg/mL. BAT lobes were injected with a total volume of 60 μL, delivered as three injections of 10 μL per lobe, using either PBS (vehicle control) or rS100A9 solution. Following injection, tissues were cultured overnight at 37 °C in a humidified incubator with 5% CO₂. Tissues were subsequently either fixed in 4% paraformaldehyde for senescence-associated β-galactosidase staining or homogenized in RIPA buffer for protein extraction and Western blot analysis.

#### CD45^+^ cells isolation and counting

Excised BAT samples were enzymatically dissociated using the Adipose Tissue Dissociation Kit (#130-105-808, Miltenyi Biotec), following the manufacturer’s protocol. Mechanical dissociation was performed using the gentleMACS™ Dissociator (Miltenyi Biotec). The resulting cell suspension was passed through a filter to eliminate residual tissue fragments and obtain a single-cell suspension. CD45⁺ cell enrichment was performed using the CD45⁺ MicroBeads Kit (#130-052-301, Miltenyi Biotec), in accordance with the manufacturer’s instructions. Briefly, cells were magnetically labeled with CD45 MicroBeads (mouse), and the suspension was applied to a MACS® Column positioned in a magnetic field provided by a MACS Separator. CD45⁺ cells were retained within the column, while unlabeled cells were eluted as the CD45⁻ fraction. To enhance purity, the retained CD45⁺ cell fraction was subjected to a second round of magnetic separation using an additional column. Following purification, CD45⁺ cells were stained with Trypan Blue and counted using Countess^TM^ Cell Counting Chamber Slides (Thermo Fisher Scientific).

#### Immunoblotting

Tissues and cells were lysed in RIPA buffer (50 mM Tris–HCl, pH 8.0, 150 mM NaCl, 12 mM deoxycholic acid, 0.5% Nonidet P-40) containing a cocktail of protease and phosphatase inhibitors (VWR Life Science® AMRESCO) and subjected to ultrasonic homogenization (3 cycles of 10 seconds each, at 40 mA). After protein quantification with the Lowry Method, sample buffer (50 mM Tris–HCl, pH 6.8, 2% SDS, 10% Glycerol, 0.02% Bromophenol blue, 1% β-mercaptoethanol) was added in a 1:1 ratio to the samples, which were boiled at 96 °C for 10 min. Next, 10 µg of proteins were loaded on SDS–PAGE under denaturing conditions (running condition 20 mA, 100 mV) and subjected to westen blot (WB) analysis.

For the analysis of proteins released into the extracellular space, conditioned media were collected to assess extracellular protein content. Samples were centrifuged at 300 × *g* for 10 min to remove dead cells. Supernatants were collected and mixed in a 1:1 ratio with sample buffer and denatured at 96 °C for 5 min. For co-culture experiments involving adipocytes and cancer cells/macrophages, the *recovery* medium described above was employed to selectively analyze proteins secreted by adipocytes only. A volume of 30 μL of conditioned medium was loaded onto the gel, and SDS-PAGE and WB were performed as previously described for cell and tissue samples.

Extracellular Vesicles (EVs) were isolated from the supernatants by the filtration/ultracentrifugation protocol as previously described [32, 33]. Medium collected from each experimental point was centrifuged at 3000 × *g* for 30 min to remove cells and debris. Supernatants were filtered through 0.22 µm filters (Millipore) and ultra-centrifuged (L7-65 Ultracentrifuge, Beckman Coulter) using a SW28 rotor, at 100,000 × *g* for 150 min at 4 ^◦^C. After ultracentrifugation EVs were resuspended in sample buffer loaded onto gel. SDS-PAGE and WB were performed as previously described for cell and tissue samples.

For the WB analyses, membranes were blocked for 1 h at room temperature on a rocking platform in 5% non-fat dried milk in T-TBS buffer (24.7 mM Tris Base, 2.7 mM KCl, 0.1% TWEEN-20). Nitrocellulose membranes were incubated overnight at 4 °C with the following primary antibodies at 1:1000 dilution: AMPKα (#2603, Cell Signaling Technology), ATGL (#2439, Cell Signaling Technology), TUBB (#10094-1-AP, Proteintech), CD15 (cat. no. sc-53290, Santa Cruz Biotechnology), HSL (cat. no. 4107, Cell Signaling Technology), NF-κB p65 (#4764, Cell Signaling Technology), PLIN1 (#sc-67164, Santa Cruz Biotechnology), Phospho-AMPKα (Thr172) (#2535, Cell Signaling Technology), Phospho-HSL (S563) (#4139, Cell Signaling Technology), Phospho-HSL (S660) (#4126, Cell Signaling Technology), Phospho-NF-κB p65 (S536) (#3033, Cell Signaling Technology), Phospho-p38 MAPK (Thr180/Tyr182) (#4511, Cell Signaling Technology), Phospho-(Ser/Thr) PKA Substrate (#9621, Cell Signaling Technology), p38 MAPK (#9212, Cell Signaling Technology), S100A8 (#47310, Cell Signaling Technology), S100A9 (#73425, Cell Signaling Technology), UCP1 (#ab23841, Abcam), VINC (#MA5-11690, Thermo Fisher Scientific). After three washes in T-TBS, membranes were incubated for 1 h at room temperature with HRP-conjugated secondary antibodies diluted 1:3000. Immunoreactive bands were detected using chemiluminescent HRP substrates (luminol and hydrogen peroxide, Bio-Rad Laboratories) and imaged with the ChemiDoc Imaging System (Bio-Rad Laboratories). Band intensity was quantified using ImageJ software and normalized to loading controls.

#### RNAseq processing and analysis

Total RNAs from cells and tissues were extracted using Direct-zol^TM^ RNA MiniPrep (ZYMO RESEARCH) according to the manufacturer’s instructions. Total RNA was quantified using the Qubit 4.0 fluorimetric Assay (Thermo Fisher Scientific). Libraries were prepared from 50 ng of total RNA using the NEGEDIA Digital mRNA-seq research grade sequencing service (Next Generation Diagnostic srl), which included library preparation, quality assessment, and sequencing on a NovaSeq 6000 sequencing system using a single-end, 75-cycle strategy (Illumina Inc.).

The RNAseq data were quality-checked using FastQC v0.11.9 and subsequently aligned to the Gencode mouse reference genome (mm10) using Hisat2 v2.2.1 [34] with default parameters. The number of reads for all RefSeq genes was counted using feature Counts v2.0.3 [35] using the multi-mapping option.

The resulting count matrix was analyzed in R using the DESeq2 package v1.40.2 [36] and Differential Expression was determined using a cutoff significance of padj <0.05. The Gene Set Expression Analysis was made with Cluster Profiler v4.8.3. All the heatmaps were plotted using ComplexHeatmap [37, 38]. SASP score was calculated using the PathAnalyzeR score from the R package MACanalyzeR [39].

#### Untargeted proteomics

Samples preparation was done using the in StageTip (iST) method. Samples were separated by HPLC in a single run (without pre-fractionations) and analyzed by LC-MS/ MS. Instruments for LC-MS/MS analysis consisted of a NanoLC 1200 coupled via a nano-electrospray ionization source to the quadrupole-based Q Exactive HF benchtop mass spectrometer. Peptide separation was carried out according to their hydrophobicity on a home-made column, 75 mm ID, 8 Um tip, 400 mm bed packed with Reprosil-PUR, C18-AQ, 1.9 mm particle size, 120 Angstrom pore size (New Objective, Inc., cat. PF7508-250H363), using a binary buffer system consisting of solution A: 0.1% formic acid and B: 80% acetonitrile, 0.1% formic acid. Total flow rate: 300 nL/min. LC linear gradient: after sample loading, run starts at 5% buffer B for 5 min, followed by a series of linear gradients, from 5% to 30% B in 90 min, then a 10 min step to reach 50% and a 5 min step to reach 95%. This last step was maintained for 10 min. MS spectra were acquired using 3E6 as an AGC target, a maximal injection time of 20 ms, and a 120,000 resolution at 200 *m*/*z*. The mass spectrometer operated in a data dependent Top20 mode with subsequent acquisition of higher-energy collisional dissociation (HCD) fragmentation MS/MS spectra of the top 20 most intense peaks. Resolution for MS/MS spectra was set to 15,000 at 200 *m*/*z*, AGC target to 1E5, max injection time to 20 ms, and the isolation window to 1.6 Th. The intensity threshold was set at 2.0E4, and Dynamic exclusion at 30 s. All acquired raw files were processed using MaxQuant (1.6.2.10) and the implemented Andromeda search engine. For protein assignment, spectra were correlated with the Human (v. 2021), including a list of common contaminants. Searches were performed with tryptic specifications and default settings for mass tolerances for MS and MS/MS spectra. The other parameters were set as follows: fixed modifications: carbamidomethyl (C); variable modifications: oxidation, acetyl (N-term); digestion: trypsin, Lys-C; min. peptide length = 7; max. peptide mass = 470 Da; false discovery rate for proteins and peptide-spectrum = 1%.

For further analysis, the Perseus software (1.6.2.3) [40] was used and first filtered for contaminants and reverse entries, as well as proteins that were only identified by a modified peptide. The LFQ Ratios were logarithmized, grouped, and filtered for min.valid number (min. 3 in at least one group). Missing values have been replaced by random numbers that are drawn from a normal distribution. Two-sample *t*-test analysis was performed using FDR = 0.01. Proteins with difference Log2 Difference R ± 1 and *q* value < 0.01 were considered significantly enriched. Proteomics data were integrated with transcriptomics data by 2D density plot in R and ggplot2.

#### Enzyme-Linked Immunosorbent Assay (ELISA)

S100A9 levels in mouse sera and culture supernatants from mouse (mBA) and human (hBA) brown adipocytes were quantified using commercial sandwich ELISA kits (Mouse S100A9 ELISA Kit, # NBP2-62856; Human S100A9 ELISA Kit, # NBP2-62855, Novus Biologicals) according to the manufacturer’s instructions. Catecholamine (CA) levels in serum and BAT lysates from CTR and CAC mice were quantified using a competitive ELISA kit (Mouse Catecholamine (CA) ELISA Kit, Novatein Biosciences) according to the manufacturer’s protocol. Absorbance was measured at 450 nm using a Tecan Sunrise Microplate Reader, and data were analyzed using Magellan™ software (Tecan Group Ltd). Concentrations were calculated from standard curves provided with each kit.

#### qPCR (Real-Time PCR)

Total RNA was extracted using TRI Reagent® (Sigma Aldrich), and 1 μg of RNA was treated with genomic DNase and retrotranscribed using the SensiFAST™ cDNA Synthesis Kit (#BIO‑65054, Bioline) following the manufacturer’s protocol.

qPCR was performed on 50 ng of cDNA using the QuantStudio3™ Real-Time PCR System (ThermoFisher Scientific), PowerUp SYBR Green Master Mix (ThermoFisher Scientific), and 96-well optical plates (MicroAmp™ Fast Optical 96-Well Reaction Plate).

qPCR was performed in triplicate by using validated qPCR primers (BLAST) for primers mouse: *Actb* (5′ -TGCTGTCCCTGTATGCCTCTG, 5′- TGATGTCACGCACGATTTCC-3′), *Rpl8* (5′-GGAGCGACACGGCTACATTA-3′, 5′-CCGATATTCAGCTGGGCCTT-3′), *Pparg* (5′-CGCTGGGGTATTGGGTCG-3′, 5′-TTCAAATCTTGTCTGTCACACAGT-3′), *S100a9* (5′-GGAGCGCAGCATAACCACCATC-3′, 5′-GCCATCAGCATCATACACTCCTCA-3′), *Cd36* (5′-AAGGCCATCTCTACCATGCC-3′, 5’-TGTGGCTAAATGAGACTGGGAC-3′), *Ucp1* (5′-ACCACCCTGGCAAAAACAGA-3′, 5′-GAGGCAGGTGTTTCTCTCCC-3′), *Il1b* (5′-TGCCACCTTTGTGATG-3′, 5′-AAGGTCCACGAGACAC-3′), *Il6* (5′-GGATACCACTCCCAACAGACC-3′, 5′-GCCATTGCACAACTCTTTTCTCA-3′), *Tnfa* (5′-ATGGCCTCCCATCAGT-3′, 5′-CTTGGTGGTTACGACG-3’), *Nos2* (5′-GCCTTCAACACCAAGGTTGTC-3′, 5′-ACCACCAGCAGTAGTTGCTC-3′), *p16* (5′-GCCCAACGCACCGAATAGTT-3′, 5′-CACGGGTCGGGTGAGAGT-3′), *p21* (5′-GCAGAATAAAAGGTGCCACAGG-3′, 5’-AAAGTTCCACCGTTCTCGGG-3′), *Tp53* (5′-CGTGGAGTATCGTCCCGACC-3′, 5′-GCAGGGACCGGAAGTCAGTT-3′), *Col3a1* (5′-TCTGCCACCCCGAACTCAAG-3′, 5’-TCCACCAGTGCTTACGTGGG-3′), *Murf1* (5′-GACAAAGACTTGGTGTGACGC-3′, 5′-CTCCAGGTTCTCCATAGCGTT-3′), *Col6a1* (5’-AGCGTGGATGCGGTCAAGTA-3′, 5′-CATCTTCCAGACCCCCGCAT-3′), *Tlr4* (5′-GCTTGAATCCCTGCATAGAGGTAG-3′, 5′-TCTTCAAGGGGTTGAAGCTCAGA-3′), *Col6a3* (5′-CAGGTCCGCTCAGGGTTCAC-3′, 5′-AAACACTATGTCAGCCGCCG-3′), *Fabp4* (5′-AAATCACCGCAGACGACAGG-3′, 5′-TCCATCCCACTTCTGCACCT-3′), *Col1a1* (5′-GTACATCAGCCCGAACCCCA-3′, 5′-GGTGGACATTAGGCGCAGGA-3′), *Rage* (5’-AACACAGGAAGAACTGAAGCTTGG-3′, 5′-CTTTGCCATCGGGAATCAGAAGTT-3′), *Atrogin1* (5′-GCGACCTTCCCCAACGCCTG-3′, 5′-GGCGACCGGGACAAGAGTGG-3′); and for primers human: *ACTB* (5′-CACCATTGGCAATGAGCGGTTC-3′, 5′ -AGGTCTTTGCGGATGTCCACGT-3′), *TP53* (5′-TGACACGCTTCCCTGGATTG-3′, 5′-GTTTCCTGACTCAGAGGGGG-3′), *RPL8* (5′-CACCATGCCTGAGGGTACAA-3′, 5′-CGGGTCTTCTTGGTCTCAGG-3′), *P16* (5’-GCCCAACGCACCGAATAGTT-3′, 5′-CACGGGTCGGGTGAGAGT-3′), *P21* (5′-AGTCAGTTCCTTGTGGAGCC-3’, 5’-GCATGGGTTCTGACGGACAT-3′), *S100A9* (5′-TGGAGGACCTGGACACAAATG-3′, 5′-TCGTCACCCTCGTGCATCTT-3′), *S100A8* (5′ – GCTAGAGACCGAGTGTCCTCAG-3′, 5′-GCCCATCTTTATCACCAGAATG-3′), *COL3A1* (5′-AATGGTGCTCCTGGACTGCG-3′, 5′-AATACCAGCCTCACCGCGTTC-3′), *IL6* (5′-CTTCGGTCCAGTTGCCTTCT-3′, 5′-GGGCGGCTACATCTTTGGAA-3′), *NOS2* (5’-CGCATGACCTTGGTGTTTGG-3′, 5′-CATAGACCTTGGGCTTGCCA-3′),*TNFA* (5′-ATCCTGGGGGACCCAATGTA-3′, 5′-AAAAGAAGGCACAGAGGCCA-3′). mRNA levels were normalized to RPL8 mRNA, and the relative mRNA levels were determined through the 2^−ΔΔCt^ method. Two reference genes (*RPL8* and *ACTB*) were initially evaluated for RT-qPCR normalization. As both reference genes showed stable expression across experimental groups and yielded comparable normalized results, *RPL8* was used as the reference gene throughout the manuscript.

#### Immunofluorescence on cells

Cells were fixed with 4% neutral paraformaldehyde (PFA, Sigma Aldrich) for 10 min at room temperature and permeabilized with 1% Triton X-100 in PBS for 10 min, followed by three 5-minute washes with PBS-T (0.1% Tween-20 in PBS) and blocked using 1% BSA in PBS-T for 1 h at room temperature. Subsequently, cells were incubated for 18 h with primary antibody 1:100 anti-S100A9 (#73425, Cell Signaling Technology). Secondary anti-rabbit antibody conjugated to Alexa Fluor 555® (Thermo Fisher Scientific) (1:300 in 5% BSA/PBS-T) was incubated for 1 h at room temperature. Nuclear staining was carried out using 10 μg/mL Hoechst 33342 (Molecular Probes, Eugene) in ddH₂O. Images were acquired using a Zeiss Axio Vert fluorescence microscope (Carl Zeiss) and processed with ZEISS ZEN 3.11 image analysis software.

#### Immunofluorescence on tissue sections

S100A9 Immunofluorescent staining: Mouse FFPE (3 mm thickness) samples were incubated overnight with a primary anti-S100A9 antibody (#NB110-89726, rabbit anti-mouse, 1:100 titer, Novus Biological). An Alexa Fluor 488® secondary donkey anti-rabbit (Invitrogen) was added for 1 h (1:200 titer). Nuclear counterstaining was performed using Hoechst 33342 1:1000 for 30 min at room temperature. Fluorescent images were collected with a Nikon A1 confocal Laser Microscope System, original magnification 60× (Nikon). Six fields were randomly chosen for each case (original magnification 600×), and the arbitrary unit of S100A9 in adipose tissue was evaluated using Fiji software (US NIH).

#### Immunohistochemistry (IHC) analysis

Formalin-fixed, paraffin-embedded (FFPE) tissue sections (3 μm) were subjected to immunohistochemical staining for S100A9 using a rabbit anti-S100A9 primary antibody (NB110-89726, Novus Biologicals). Antigen retrieval was performed in citrate buffer (pH 6.0; Sigma-Aldrich, C9999) for 30 min at 95 °C. Sections were then incubated overnight at 4 °C with the primary antibody diluted 1:200. After washing, sections were incubated for 1 hour at room temperature with EnVision FLEX/HRP secondary antibody (Dako, cat. no. GV800). Immunoreactivity was visualized using 3,3′-diaminobenzidine (DAB) chromogen (DAKO Omnis EnVision FLEX/HRP, USA), and nuclei were counterstained with hematoxylin. Stained sections were imaged using an Olympus BX51 microscope equipped with a Micropublisher 6™ camera. For quantitative evaluation of S100A9 expression, five randomly selected fields per section were analyzed at 400× magnification. Immunostaining intensity was assessed using the open-source IHC Profiler plugin for ImageJ software.

#### Oil red-O staining

Intracellular lipid droplets were stained using the lipophilic dye Oil Red-O (ORO, Sigma Aldrich). After a 10 min cell fixation in 4% PFA, three washes in PBS and one wash in ddH₂O were performed. The stock solution (0.5% ORO in isopropanol, filtered) was dissolved in ddH_2_O in a 3:2 ratio and filtered. Adipocytes were incubated with the ORO solution for 20 min, then washed three times with PBS and once with ddH₂O. For brightfield or fluorescence microscopy, nuclei were stained with Hoechst 3334. After a wash with PBS, images were acquired using a fluorescence microscope (Zeiss Axio Vert, Thermo Fisher Scientific). For quantitative absorbance analysis, unbound ORO was first removed by washing with 60% isopropanol. Lipid-bound ORO was then eluted with 100% isopropanol, and the eluate was transferred into a 96-well microplate. Absorbance was measured at 492 nm using a Tecan Sunrise Microplate Reader, and data were analyzed using Magellan™ software (Tecan Group Ltd).

#### Histological staining

FFPE tissues explants were cut into 3 mm thickness and stained with Haematoxylin and Eosin (H&E). Three fields were randomly chosen for each case (original magnification 400×), and the diameter of about 400 intracellular fat vacuoles in adipose tissue was measured using QuPath software. Masson’s trichrome staining was performed on Mouse FFPE (3 mm thickness) to evaluate fibrosis in the adipose tissue of both CTR and CAC cases. Sections were scanned using NanoZoomer 2.0-RS (original magnification 200×). Histological images stained with Masson’s Trichrome were analyzed using ImageJ software. For each image, the area of collagen deposition (stained green) was quantified by color thresholding, followed by binary conversion and measurement of the positive area. The collagen-positive area was then expressed as a percentage of the total tissue area to assess fibrosis extent.

#### Glycerol assay

Glycerol assay was performed using a commercial kit (#MAK117, Sigma Aldrich) following the manufacturer’s instructions. Glycerol concentration was determined via a coupled enzymatic assay involving glycerol kinase and glycerol phosphate oxidase, resulting in a colorimetric product proportional to the glycerol content and measured at 570 nm using the Tecan Sunrise Microplate Reader, and data were analyzed using the Magellan™ software (Tecan Group Ltd).

#### Oxygen consumption test

Oxygen consumption was measured in brown adipocytes using the Oxygraph Plus oxygen electrode system (Hansatech Instruments Ltd., Norfolk, UK). Cells were resuspended at 1 × 10⁶ cells/mL in culture medium without FBS to assess basal oxygen consumption and were stimulated with 1 µM FCCP to determine maximal oxygen consumption. Oxygen consumption rates were recorded at 37 °C for 10 min and normalized to protein concentration.

#### Senescence-associated β-galactosidase (SA-β-gal) assay

Senescence-associated β-galactosidase (SA-β-gal) activity in cultured brown adipocytes and BAT sections was assessed using the *Senescence Cells Histochemical Staining Kit* (#CS0030, Sigma Aldrich), which is based on a histochemical detection method at pH 6, following the manufacturer’s instructions. For tissue analysis, BAT was explanted, embedded in optimal cutting temperature (OCT) compound, and snap-frozen. Tissue blocks were sectioned at 12 μm using a Histoline MC5050 cryostat microtome and mounted onto poly-D-lysine–coated glass slides (Thermo Fisher Scientific). Sections were subjected to the SA-β-gal assay and mounted with DPX mounting medium. SA-β-gal–positive senescent cells (dark blue staining) were visualized by bright-field microscopy using a Zeiss Axio Vert microscope (Carl Zeiss). Images were acquired and processed using ZEISS ZEN 3.11 software.

#### Statistics

Animal sample size was determined with an a priori power calculation using G* Power analysis. For in vitro analyses, the exact numbers of replicates are given in each figure and were sufficient to detect biologically meaningful differences. Investigators were not blinded to group allocation during the experiments, but biases were minimized where possible. Data distribution normality was assessed using the Shapiro–Wilk test implemented in GraphPad Prism 10 before performing parametric statistical analyses. A two-tailed unpaired Student’s *t*-test was performed to assess the statistical significance between two groups. Analysis of variance (ANOVA) followed by Tukey’s (multiple comparisons among groups) post hoc tests was used to compare three or more groups. Venn diagrams were constructed using Venny 2.1.0 software. Statistical analyses were performed using GraphPad Prism 10 (GraphPad Software Inc.).

### Ethics approval and consent to participate

All methods were performed in accordance with the relevant guidelines and regulations. The research was approved by relevant local (The University Animal Welfare Committee-OPBA, Tor Vergata University) and national (Ministry of Health, Legislative Decree No. 26/2014; European Directive 2010/63/UE) committees with authorization n° 27/2021-PR. Animal experiments were conducted according to guidelines approved by the University Animal Welfare Committee-OPBA and Ministry of Health.

## Results

### Characterization of BAT phenotype in a mouse model of cancer-associated cachexia

**Fig. 1:**
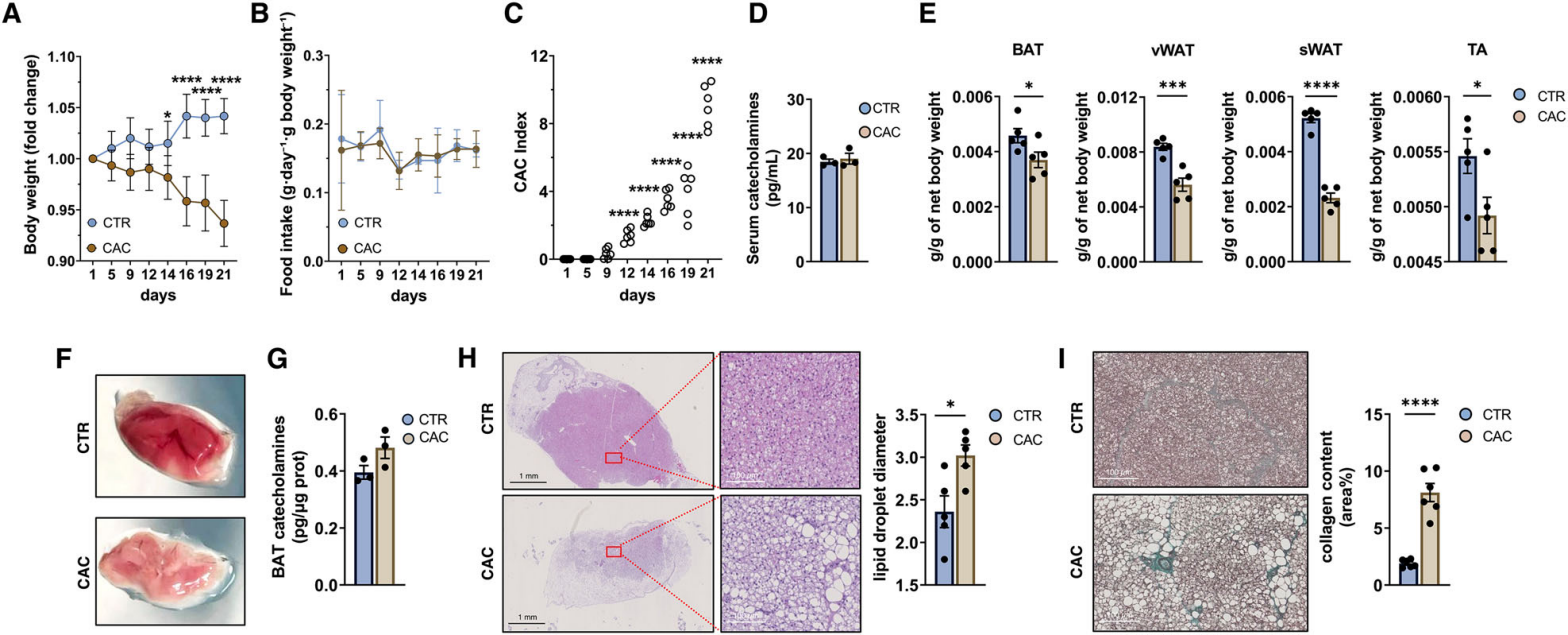
Cancer cachexia induces progressive weight loss and histological remodeling of brown adipose tissue. **A** Body weight trajectory in control (CTR) and LLC tumor-bearing cachectic mice (CAC) over time. Data are mean ± SD (*n* = 6 per group; unpaired two-tailed *t*-test; *p < 0.05, ****p < 0.0001). **B** Food intake in CTR and CAC mice over time. Data are mean ± SD of daily food intake normalized to body weight (*n* = 6 per group; unpaired two-tailed *t*-test; *p < 0.05, ****p < 0.0001). **C** Cachectic (CAC) index analysis (*n* = 6 per group; unpaired two-tailed *t*-test; ****p < 0.0001). **D**, Catecholamine levels in sera of CTR and CAC mice. Data are mean ± SD (*n* = 3 per group; unpaired two-tailed *t*-test). **E** Tissue weights of interscapular BAT, visceral (vWAT), subcutaneous (sWAT) adipose tissue and *tibialis anterior* (TA) in CAC mice compared to CTR. Data are mean ± SD (*n* = 5 per group; unpaired two-tailed *t*-test; *p < 0.05, ***p < 0.001, ****p < 0.0001*)*. **F** Gross morphology of BAT from CTR and CAC mice. Representative images (*n* = 6). **G** Catecholamine levels in BAT lysates of CTR and CAC mice. Data are mean ± SD (*n* = 3 per group; unpaired two-tailed *t*-test; *p* = ns). **H** Representative H&E staining (*left panel*) and lipid droplet measurement (*right panel*) in BAT from CTR and CAC mice. Scale bars, 1 mm (overview) and 100 µm (inset). Data are mean ± SD (*n* = 5 per group; unpaired two-tailed *t*-test; *p < 0.05). **I** Representative Masson’s trichrome staining (*left panel*) and quantification of fibrosis (green area; *right panel*). Scale bars, 1 mm. Data are mean ± SD (*n* = 5 per group; unpaired two-tailed *t*-test; ****p < *0.0001)*.

To explore the effects of CAC on BAT, we utilized a mouse model wherein CAC was induced by the injection of Lewis Lung Carcinoma (LLC) cells. Figure 1A illustrates the trajectory of net body weight loss in CAC compared to control (CTR) mice. A statistically significant reduction in body weight was observed starting 14 days post-injection, with weight loss continuing progressively throughout the experimental period without changes in food intake (Fig. 1B). By the end of the study, the CAC mice exhibited an average body weight reduction of approximately 8–10% with respect to CTR group. This significant decrease in body weight is a hallmark of CAC and confirms the effectiveness of the LLC model in replicating cachexia.

CAC Index was calculated as indicator of the severity of tumor-related weight loss that revealed significant cachexia occurrence without significant changes in serum catecholamine levels (Fig. 1C, D). To assess the impact of cachexia on adipose tissue and muscle mass, a comparative analysis was conducted between CAC and CTR mice. This analysis revealed a significant reduction in the weight of all examined adipose and muscle tissues in the CAC group (Fig. 1E).

BAT of CAC mice exhibited a phenotypical change that manifests as significant whitening that was not accompanied by changes in tissue catecholamine levels indicative of unaffected adrenergic stimulation (Fig. 1F–H). Detailed analyses on histological sections of BAT stained with H&E show morphology at the cellular level, with CAC mice displaying BAT with parenchyma surrounded by adipocytes with unilocular lipid droplets typical of WAT (Fig. 1H). Moreover, the BAT core is composed of multilocular brown adipocytes with larger lipid droplets than CTR BAT, indicative of a loss of BAT identity and functionality. A fibrotic remodeling was also found in CAC BAT, as visualized by the intense Masson’s trichrome green staining throughout the tissue, representing collagen deposition (Fig. 1I).

**Fig. 2:**
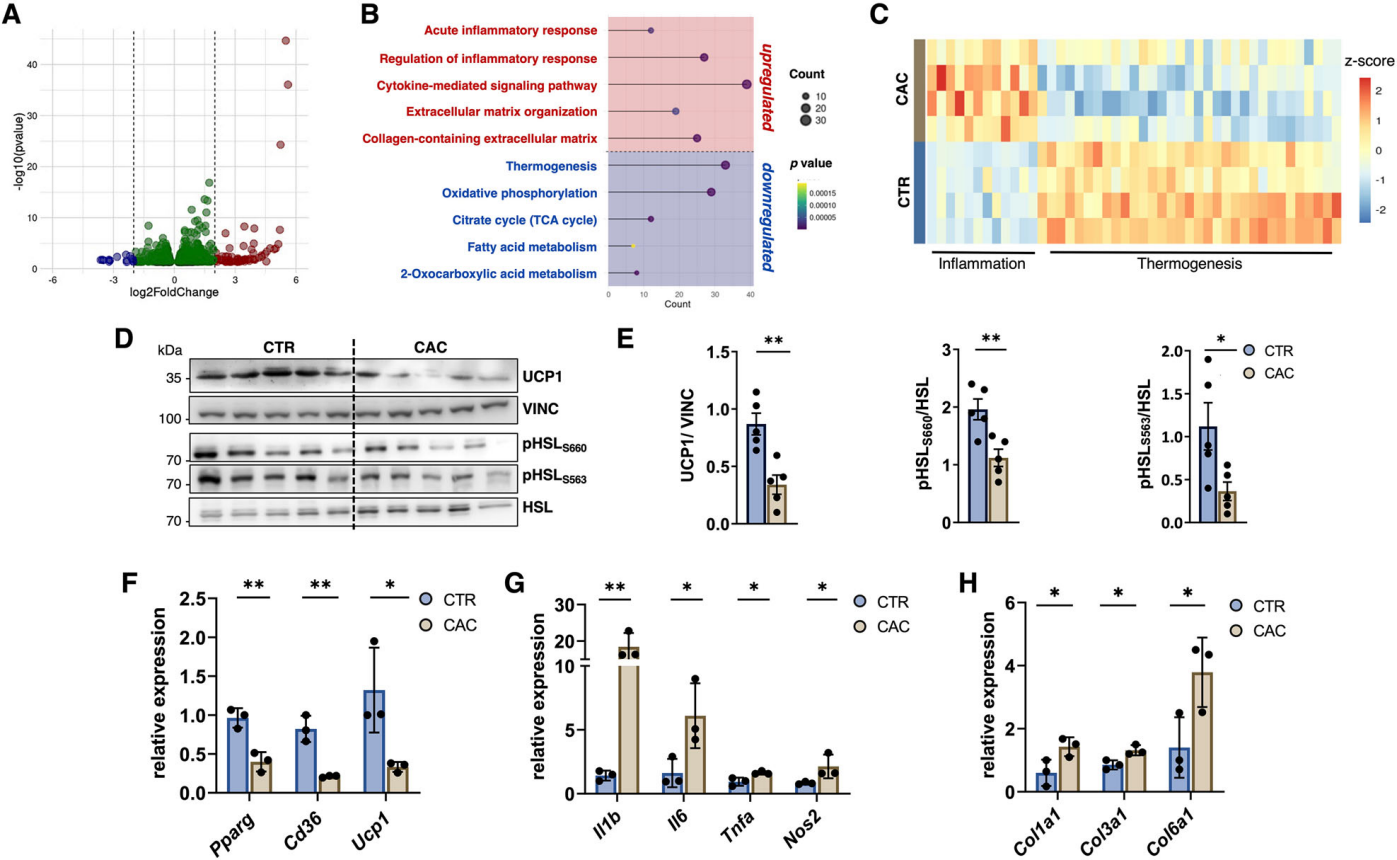
Transcriptomic and proteomic reprogramming of BAT reveals impaired thermogenic and lipolytic activity in cancer-associated cachexia. **A** Volcano plot of differentially expressed genes (DEGs) in BAT from control (CTR) and LLC-bearing cachectic (CAC) mice (*n* = 4 per group). Upregulated genes in red, downregulated in blue (*p* < 0.05). **B** Gene Set Enrichment for Gene Ontology (GO) Biological Process and KEGG terms of upregulated (red) and downregulated (blue) genes of CAC vs CTR BAT (*p* value < 0.05; log2FC < −0.5 and log2FC > 0.5). **C** Heatmap of selected inflammatory and thermogenic genes in BAT from CTR and CAC mice (*n* = 4 per group). **D** Immunoblots of UCP1, total HSL, and phospho-HSL (S660 and S563) in BAT lysates. Vinculin (VINC) was used as loading control (*n* = 5 per group). **E** Densitometry analysis of immunoblots. Data are mean ± SD (*n* = 5 per group; unpaired two-tailed *t*-test; *p < 0.05, **p < 0.01). **F–H** Relative mRNA expression of thermogenic (*Pparg, Ucp1*, *Cd36*), inflammatory (*Il1b*, *Il6*, *Tnfa*, *Nos2*), and collagen (*Col1a1*, *Col3a1*, *Col6a1*) genes in BAT from CTR and CAC mice. Data are mean ± SD (*n* = 5 per group; unpaired two-tailed *t*-test; *p < 0.05, **p < 0.01).

Following the quantification of tissue mass and assessment of overall body weight changes, we conducted a detailed molecular analysis to further elucidate the impact of LLC injection on BAT. Hence, we performed an RNAseq analysis that revealed 963 differentially expressed genes (DEGs), with 605 significantly upregulated (*p* < 0.05) (Figs. 2A and S1A, B). This substantial transcriptional shift underscores the remarkable plasticity of BAT in response to cachectic stimuli. Gene Set Enrichment Analysis (GSEA) of DEGs highlighted several key biological processes, among which inflammation-related terms, indicative of heightened tissue inflammation (Fig. 2B, C). Additionally, extracellular matrix-related terms were enriched, suggesting an increased secretory phenotype within the BAT (Fig. 2B). Conversely, terms associated with thermogenesis and metabolism, particularly oxidative phosphorylation and lipid metabolism, were significantly depleted (Fig. 2B, C). This downregulation points to substantial tissue dysfunctionality, consistent with the observed “whitening” or loss of BAT characteristics in CAC. Complementary proteomic analysis of BAT lysate corroborated RNAseq data and identified 413 differentially represented proteins, 389 of which were upregulated and primarily associated with inflammatory and secretory pathways (Fig. S1C–F).

We then validated RNAseq and proteomic data, examining the expression of key thermogenic and lipid metabolic genes. Western blot demonstrated a pronounced decrease in UCP1 levels, a critical marker of BAT thermogenic activity, in the CAC group compared to controls, confirming the loss of thermogenic capacity in BAT during cachexia (Fig. 2D, E). Hormone-sensitive lipase (HSL) and its phosphorylated active forms on Ser660 and Ser563 (pHSL_S660_, pHSL_S563_), key regulators of lipolysis, were also analyzed. While total HSL levels were unchanged, pHSL_S660_, pHSL_S563_ were markedly reduced in the CAC group, indicating a decrease in lipolytic activity (Fig. 2D, E). The qPCR analysis further elucidated the transcriptional changes in BAT during CAC. Specifically, *Ucp1* mRNA levels were significantly downregulated, consistent with the protein data, confirming the diminished thermogenic capacity at the transcriptional level (Fig. 2F). *Pparg*, a transcription factor promoting the expression of thermogenic genes, and *Cd36* promoting lipid uptake, were significantly reduced, further indicating impaired thermogenesis and lipid metabolism (Fig. 2F).

Also, we validated data indicating the onset of inflammatory and fibrotic state by analyzing some of their mediators by qPCR. These analyses confirmed that the cachectic BAT underwent increased expression of *Il1β*, *Il6*, *Tnfa,* and *Nos2* as well as upregulation of collagen genes (*Col1a1*, *Col3a1*, *Col6a1*) (Fig. 2G, H).

### Analysis of senescence-associated secretory phenotype and pro-cachectic actions of BAT during cancer-associated cachexia

**Fig. 3:**
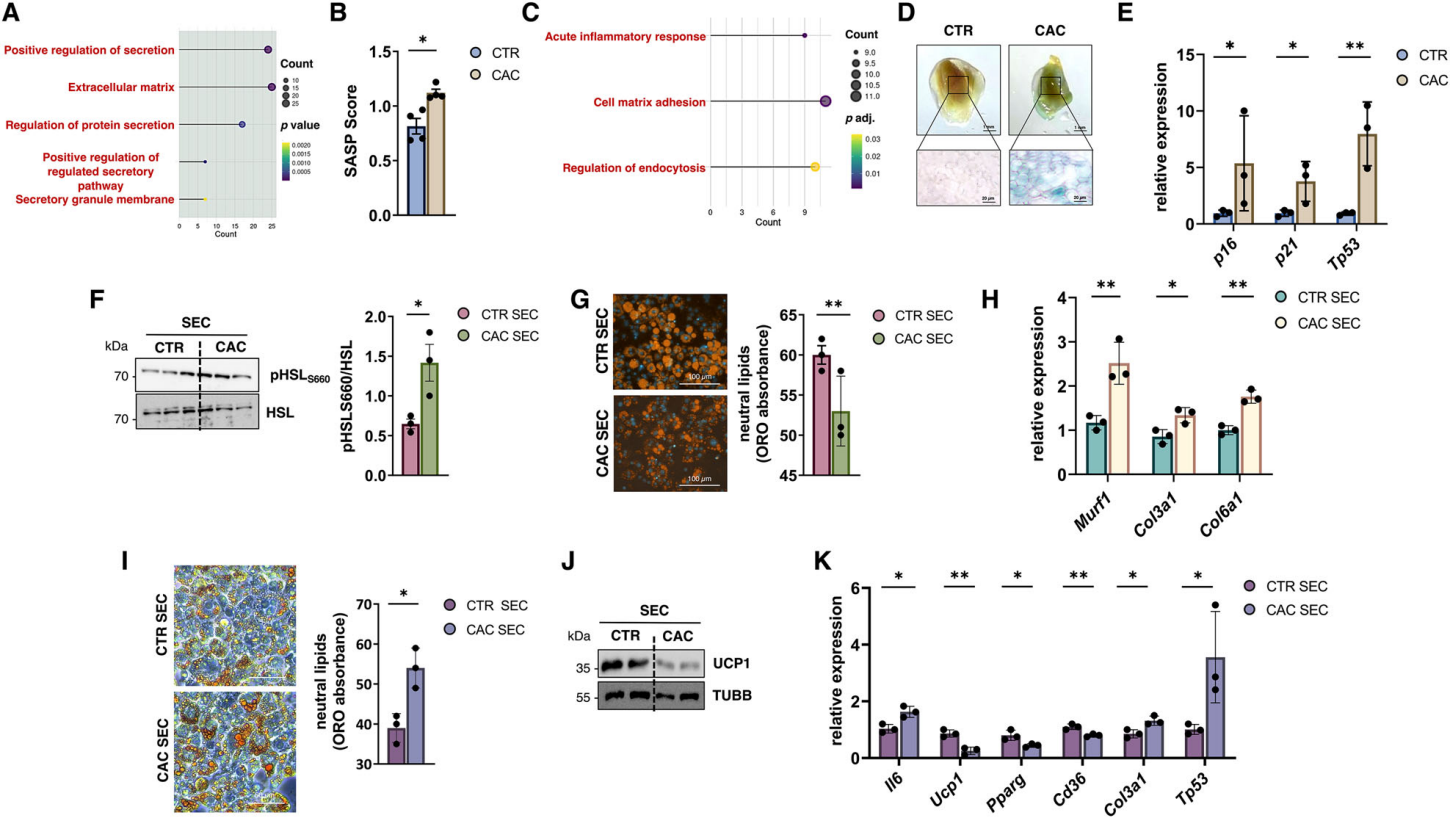
Senescence-associated phenotype and secretory activity of BAT during cancer cachexia. **A** Gene Set Enrichment Analysis (GSEA) for Gene Ontology (GO) Cellular Components of upregulated genes from RNAseq in BAT of CAC vs CTR mice (*p* value < 0.05; log2FC > 0.5). **B** Enrichment of WikiPathway “Senescence-Associated Secretory Phenotype (SASP)” in CAC BAT vs CTR. Data are mean ± SD (*n* = 4 per group; unpaired two-tailed *t*-test; *p < 0.05). **C** GSEA for GO Biological Process of upregulated proteins in BAT secretome from CAC mice vs CTR (*n* = 4 per group). **D** Representative SA-β-gal staining in BAT from CTR and CAC mice. Scale bars, 2 mm. Insets show representative micrograph images of tissue sections after SA-β-gal staining. Scale bars, 20 µm (*n* = 6 per group). **E** Relative mRNA levels of senescence markers (*p16*, *p21*, *Tp53*) in BAT from CTR and CAC mice. Data are mean ± SD (*n* = 3 per group; unpaired two-tailed *t*-test; *p < 0.05, **p < 0.01). **F** Immunoblot (*left panel*) and quantification (*right panel*) of phospho-HSL (S660) in 3T3-L1 adipocytes treated with BAT secretome (SEC) from CTR or CAC mice. Data are mean ± SD (*n* = 3; unpaired two-tailed *t*-test; *p < 0.05). **G**, Oil Red O staining (*left panel*) and quantification of lipid content (*right panel*) in 3T3-L1 white adipocytes after SEC exposure. Scale bars, 100 µm. Data are mean ± SD *(n* = 3; unpaired two-tailed *t*-test; **p < 0.01). **H** Relative mRNA expression of atrophy-related genes (*Murf1*, *Col3a1*, *Col6a1*) in C2C12 myotubes treated with BAT SEC. Data are mean ± SD (*n* = 3; unpaired two-tailed *t*-test; *p < 0.05, **p < 0.01). **I** ORO staining (*left panel*) and quantification of lipid droplets (*right panel*) in T37i brown adipocytes exposed to BAT SEC. Scale bars, 50 µm. Data are mean ± SD (*n* = 3; unpaired two-tailed *t*-test; *p < 0.05). **J** Representative immunoblot of UCP1 protein from T37i brown adipocytes after BAT SEC exposure. Tubulin (TUBB) staining was used as loading control. **K** Relative mRNA expression levels of *Il6*, *Ucp1*, *Pparg*, *Cd36, Col3a1, Tp53* in T37i brown adipocytes exposed to BAT SEC. Data are mean ± SD (*n* = 3; unpaired two-tailed *t*-test; *p < 0.05, **p < 0.01).

The data described thus far indicate that the loss of thermogenic function in BAT during cachexia is accompanied by the upregulation of inflammatory processes and fibrotic remodeling. In addition, RNAseq analysis showed a significant enrichment of the secretion pathways and extracellular matrix (Fig. 3A). Notably, the combined features of inflammatory cytokine production, collagen deposition, mitochondrial dysfunction, and adipocyte hypertrophy observed in cachectic BAT closely recapitulate a Senescence-Associated-Secretory Phenotype (SASP) [41], resulting in an elevated SASP score in CAC mice compared to CTR (Fig. 3B). To provide more conclusive evidence for a secretory phenotype, BAT explants were cultured ex vivo, and secreted proteins (secretome) collected for proteomic analysis. This analysis identified 370 differentially represented proteins, with 290 being significantly upregulated in the secretome of CAC mice (Fig. S1G–I). Functional enrichment revealed that these secreted proteins pertain to “acute inflammatory response” (Fig. 3C), substantiating RNAseq and proteomic data on BAT lysates.

To confirm that BAT undergoes senescence during CAC, we initially performed Senescence-Associated β-galactosidase (SA-β-gal) staining. BAT from CAC mice displayed intense blue staining in adipocytes (Fig. 3D)—indicative of cellular senescence—which was completely absent in control BAT. Key senescence-associated genes, including *p16* and *p21*, as well as the senescence regulator *Tp53*—previously implicated in adipose tissue dysfunction [42] —were significantly upregulated at the mRNA level (Fig. 3E), consistent with increased SASP score.

Next, we assessed whether the BAT secretome could elicit cachectic-like effects in target white adipose and skeletal muscle cells. To this end, secretomes from ex vivo cultured BAT of CTR or CAC mice were applied to cultured white adipocytes and skeletal muscle myotubes. Significant alterations were observed in both cell types. Specifically, in white adipocytes, we detected a marked increase in phospho-active HSL (Fig. 3F), accompanied by a reduction in intracellular lipid content (Fig. 3G), arguing that lipolysis was induced. In myotubes, the CAC secretome evoked a pro-atrophic response, characterized by a significant upregulation of key atrophy-related genes, including *Murf1* and collagen genes (*Col3a1*, *Col6a1*) compared to the CTR secretome (Fig. 3H).

We then hypothesized that the pathological remodeling of BAT observed in CAC might also involve an autocrine mechanism mediated by its own secretome. To test this, brown adipocytes were treated with BAT-derived secretome, following the same experimental approach used for white adipocytes and myotubes. Brown adipocytes exposed to the CAC secretome exhibited a marked accumulation of intracellular lipid droplets and a reduction in UCP1 protein levels compared to those treated with CTR secretome (Fig. 3I, J). In parallel, gene expression analysis revealed a significant downregulation of transcripts involved in lipid uptake (*Cd36*) and thermogenesis (*Ucp1* and *Pparg*), together with an upregulation of inflammatory (*Il6*), senescence (*Tp53*), and fibrotic (*Col3a1*) markers (Fig. 3K).

These findings closely recapitulate the molecular alterations observed in vivo in cachectic BAT and support the notion that tumor burden reshapes the BAT secretome, promoting the release of pro-cachectic factors that trigger skeletal muscle cell atrophy, white adipocyte lipolysis, and the loss of thermogenic identity in brown adipocytes.

**Fig. 4:**
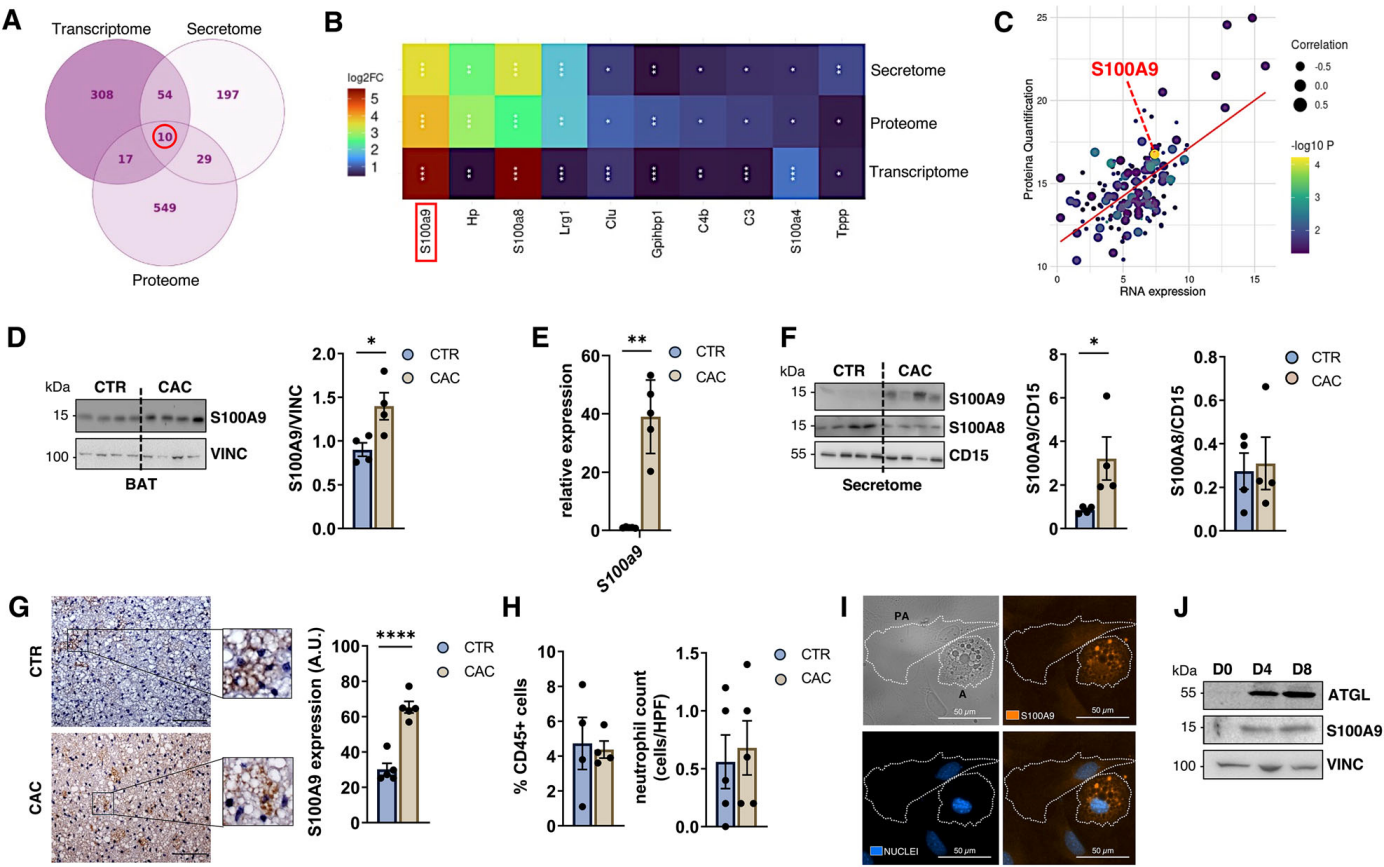
Brown adipocytes are the primary source of S100A9 in cachectic BAT and actively secrete it in response to lung cancer signals. **A** Venn diagram of commonly upregulated genes and proteins in BAT transcriptome, proteome, and secretome from CAC mice (*n* = 4 per group). **B** Heatmap of the 10 common upregulated genes/proteins in all three datasets (transcriptome, proteome and secretome) in BAT from CAC mice (*n* = 4 per group; one way ANOVA test; *p < 0.05, **p < 0.01, ***p < 0.001). **C** Correlation between RNA and protein expression in BAT samples from CAC mice. S100A9 is highlighted as the top multi-omic correlate. **D** Immunoblot (*left panel*) and quantification (*right panel*) of S100A9 in BAT lysates from CTR and CAC mice. Vinculin (VINC) was used as loading control. Data are mean ± SD (*n* = 4; unpaired two-tailed *t*-test; *p < 0.05). **E** Relative mRNA expression of S100a9 in BAT from CTR and CAC mice. Data are mean ± SD (*n* = 5; unpaired two-tailed *t*-test; **p < 0.01). **F** Immunoblot (*left panel*) and quantification (*right panel*) of S100A9 and S100A8 in BAT secretome from CTR and CAC mice. CD15 was used as loading control. Data are mean ± SD (*n* = 4; unpaired two-tailed *t*-test; *p < 0.05). **G** Representative immunohistochemistry images (*left panel*) and quantification of S100A9 protein levels (*right panel*) in BAT sections from CTR and CAC mice. Insets show representative high-magnification details of S100A9-positive brown adipocytes from both CTR and CAC groups. Original magnification, 40×. Scale bar, 50 μm. Data are mean ± SD (*n* = 5; unpaired two-tailed *t*-test; ****p < 0.0001). **H** Quantification of CD45⁺ cells in BAT stromal vascular fraction (*left panel*) and quantification of neutrophils on H&E stained sections (*right panel*) from CTR and CAC BAT. Neutrophils were quantified in six non-overlapping high-power fields (HPF) per section at 40× magnification. Data are mean ± SD (*n* = 4 and 5, respectively; unpaired two-tailed *t*-test; *p* = ns). **I** Representative immunofluorescence staining of S100A9 (red) in undifferentiated and differentiated immortalized murine brown adipocytes (mBA). Scale bars, 50 µm (*n* = 3). PA Pre-Adipocytes, A Adipocytes. **J** Representative immunoblot of S100A9 and ATGL from undifferentiated (D0, day 0), differentiating (D4, day 4), and differentiated (D8, day 8) mBA brown adipocytes. Vinculin (VINC) was used as loading control.

In the context of CAC, we sought to identify key molecular factor(s) released by BAT that could majorly contribute to the observed alterations in treated myotubes as well as white and brown adipocytes. Integration of transcriptomic, proteomic, and secretomic data revealed ten commonly upregulated factors across all three datasets (Fig. 4A). This comprehensive analysis identified S100A9 as one of the most substantially upregulated in BAT of CAC mice (Fig. 4B) with a very strong correlation between gene and protein expression (Fig. 4C).

This S100A9 upregulation was further validated and characterized through a series of molecular and histological assessments. S100A9 was found to be significantly upregulated in CAC BAT at both protein and mRNA levels (Fig. 4D, E). In ex vivo–cultured BAT, this secreted protein was markedly elevated in CAC samples, as shown by increased band intensity in immunoblots (Fig. 4F), confirming the secretomic data. Notably, in contrast to S100A9, S100A8 protein was only minimally detected and did not show appreciable differences between groups (Fig. 4F). The increased release of S100A9 was also observed systemically, with higher levels detected in mouse plasma of CAC than CTR mice (Fig. S2A). Likewise, most of the BAT-secreted S100A9 was found in the soluble fraction, while only minimal amounts were associated with Extracellular Vesicles (Fig. S2B).

S100A9 is canonically described as an alarmin secreted by phagocytic immune cells in response to tissue damage and inflammatory stress, including cancer [43, 44]. Hence, we sought to determine the cellular source of S100A9 in our model. Immunohistochemistry and immunofluorescence staining of S100A9 in BAT sections revealed the presence of S100A9 within brown adipocytes in CTR mice, and strongly elevated levels within brown adipocytes of CAC mice (Figs. 4G and S2C). By contrast, S100A9 was expressed at low levels in WAT and did not undergo elevation in CAC WAT compared to control group (Fig. S2D).

Moreover, immunohistochemistry on BAT sections revealed no differences in S100A9 staining of neutrophils, fibroblasts, and endothelial cells between CTR and CAC BAT (Fig. S2E), indicating the specific S100A9 increase in brown adipocytes. To further rule out the contribution of immune cells in S100A9 elevation, the number of CD45+ cells and neutrophils was quantified. Such analysis revealed no significant difference between CTR and CAC groups (Fig. 4H). Given that adipocytes constitute the predominant cell population within BAT, collectively, the data suggest that most of the identified secreted S100A9 protein in CAC BAT originates from brown adipocytes. Notably, both immunofluorescence and western blot analysis demonstrated that S100A9 is not present in undifferentiated murine primary brown adipocyte precursors and only becomes detectable in mature adipocytes, giving further support to the evidence that S100A9 is a protein expressed by brown adipocytes (Fig. 4I, J).

### In vitro validation of S100A9 secretion by mouse and human brown adipocytes in response to lung cancer

**Fig. 5:**
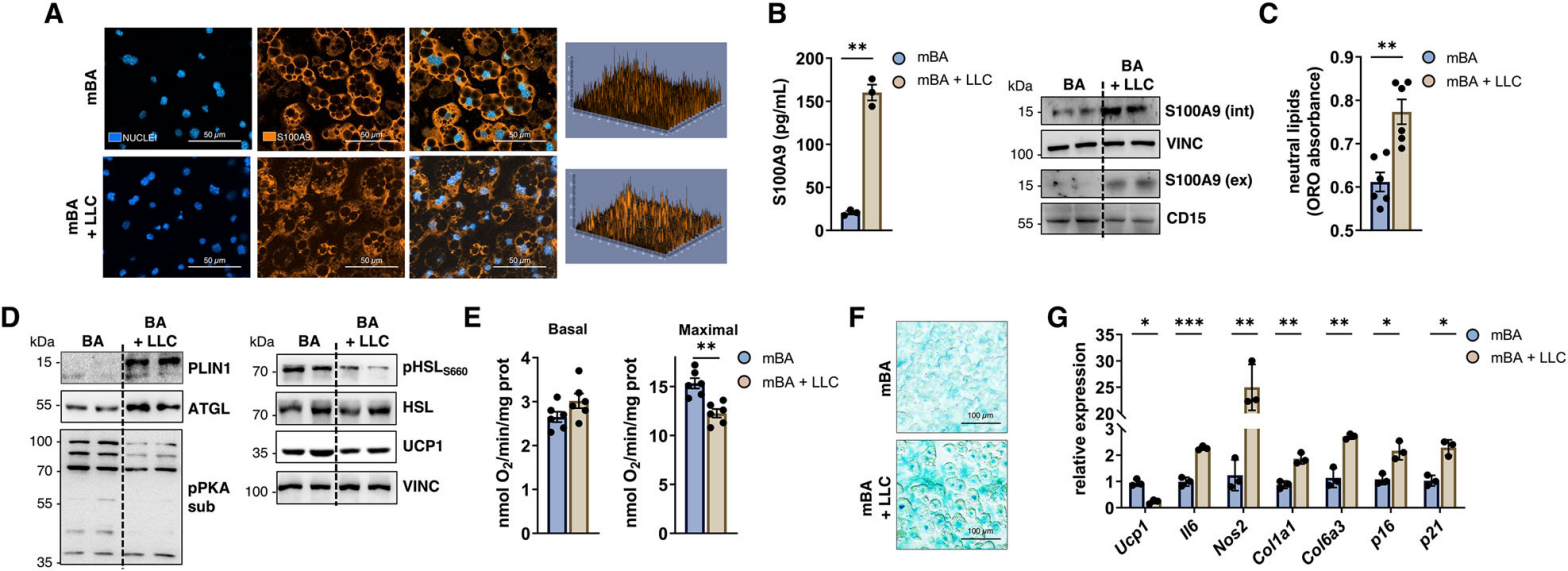
Co-culture with lung cancer cells induces S100A9 secretion and senescence-like remodeling in brown adipocytes. **A** Representative immunofluorescence staining of S100A9 (red) in immortalized murine brown adipocytes (mBA) under control conditions and after co-culture with LLC, followed by *recovery* in fresh medium. Nuclei are stained with Hoechst 33342 (blue). Right panels show 3D surface plots of fluorescence intensity distribution, representing intracellular localization and abundance of S100A9. Scale bars, 50 µm (*n* = 3). **B** ELISA quantification of S100A9 in culture supernatants from murine brown adipocytes (mBA) under control conditions and after co-culture with A549 lung cancer cells, followed by removal of cancer cells and co-culture medium and *recovery* in fresh medium prior to supernatant collection (*left panel*). Data are presented as mean ± SD (*n* = 3 per group; unpaired two-tailed *t*-test; *p < 0.05). Representative immunoblot of S100A9 in cell lysates (int) and culture media (ex) of T37i brown adipocytes (BA) after co-culture with LLC cells, collected following cancer cell removal and medium refresh. Vinculin (VINC) and CD15 were used as loading control. Data are mean ± SD (*n* = 6; unpaired two-tailed *t*-test; **p < 0.01). **C** Oil Red O quantification of lipid accumulation in mBA brown adipocytes following LLC co-culture. Data are mean ± SD (*n* = 6; unpaired two-tailed *t*-test; **p < 0.01). **D** Representative immunoblot of PLIN1, ATGL, PKA phospho-substrates (pPKA sub) (*left panel*), phospho-active HSL (Ser660), and UCP1 (*right panel*) in T37i BA with or without LLC exposure. Vinculin (VINC) was used as loading control. **E** Representative SA-β-gal staining in mBA brown adipocytes CTR, and cultured with or without LLC cells. Scale bars, 100 µm. **F** Relative mRNA expression of *Ucp1*, *Il6*, *Nos2*, *Col1a1, Col6a3, p16*, *p21*, in mBA brown adipocytes exposed to LLC cells. Data are mean ± SD (*n* = 3; unpaired two-tailed *t*-test; *p < 0.05, **p < 0.01, ***p < 0.001). **G** Cellular basal and maximal oxygen consumption determined through a polarographic method (*n* = 6, unpaired two-tailed *t*-test; *p* = ns).

To further validate the in vivo findings and determine whether brown adipocytes actively secrete S100A9 in response to cancer-derived cues, we established an in vitro model based on transient co-culture of murine brown adipocytes with LLC lung cancer cells. After the co-culture period, LLC cells and the co-culture medium were completely removed, brown adipocytes were washed, and incubated in fresh medium (*recovery*), which was subsequently used for secretome analyses. Immunofluorescence images in Fig. 5A confirmed cytoplasmic localization of S100A9 in brown adipocytes under basal conditions, while exposure to LLC cells induced a more punctate distribution pattern, consistent with active secretion. Western blot analysis of brown adipocytes showed that S100A9 protein was increased intracellularly upon LLC exposure. Importantly, ELISA and western blot analysis performed in supernatants collected exclusively during the recovery phase demonstrated evidence that S100A9 underwent extracellular accumulation (Fig. 5B), further supporting the evidence that a significant portion of S100A9 found in the BAT secretome originates from brown adipocytes in CAC mice. Co-culture with LLC, in line with what observed in vivo in BAT, led to the increase of intracellular lipids in brown adipocytes (Fig. 5C). Accordingly, Perilipin 1 (PLIN1), a protein that coats lipid droplets and inhibits lipolysis, showed an increased level in co-culture with LLC cells (Fig. 5D). Additionally, the levels of adipose triglyceride lipase (ATGL), a lipolytic enzyme residing on lipid droplets, were also increased in brown adipocytes exposed to the mimicry of cachectic environment, suggestive that the accumulation of intracellular lipids is due to impaired activation of lipolysis (Fig. 5D). Indeed, the overall levels of Protein Kinase A (PKA) phospho-substrates (pPKA sub) were significantly reduced concomitant to decreased phosphorylated active form of hormone-sensitive lipase (pHSL_S660_) (Fig. 5D). Notably, also UCP1 protein was significantly decreased in association with reduced maximal oxygen consumption (Fig. 5D, E), indicating impaired mitochondrial respiratory capacity and reduced ability to increase oxidative metabolism under uncoupling conditions. As for in vivo, increased positivity to SA-β-gal staining was observed upon LLC co-culture (Fig. 5F), suggesting the development of SASP. Accordingly, besides the confirmed *Ucp1* mRNA downregulation, a significant upregulation of senescence (*p16*, *p21*), inflammatory (*Il6, Nos2*), and fibrotic (*Col1a1*, *Col6a3*) markers were observed (Fig. 5G).

A comparable inflammatory and senescent phenotype was observed when brown adipocytes were co-cultured with LPS-stimulated RAW264.7 macrophages, mirroring the effects induced by LLC cells. This included increased secretion of S100A9 accompanied by upregulation of *Il6* and senescence markers (Fig. S2F–H).

**Fig. 6:**
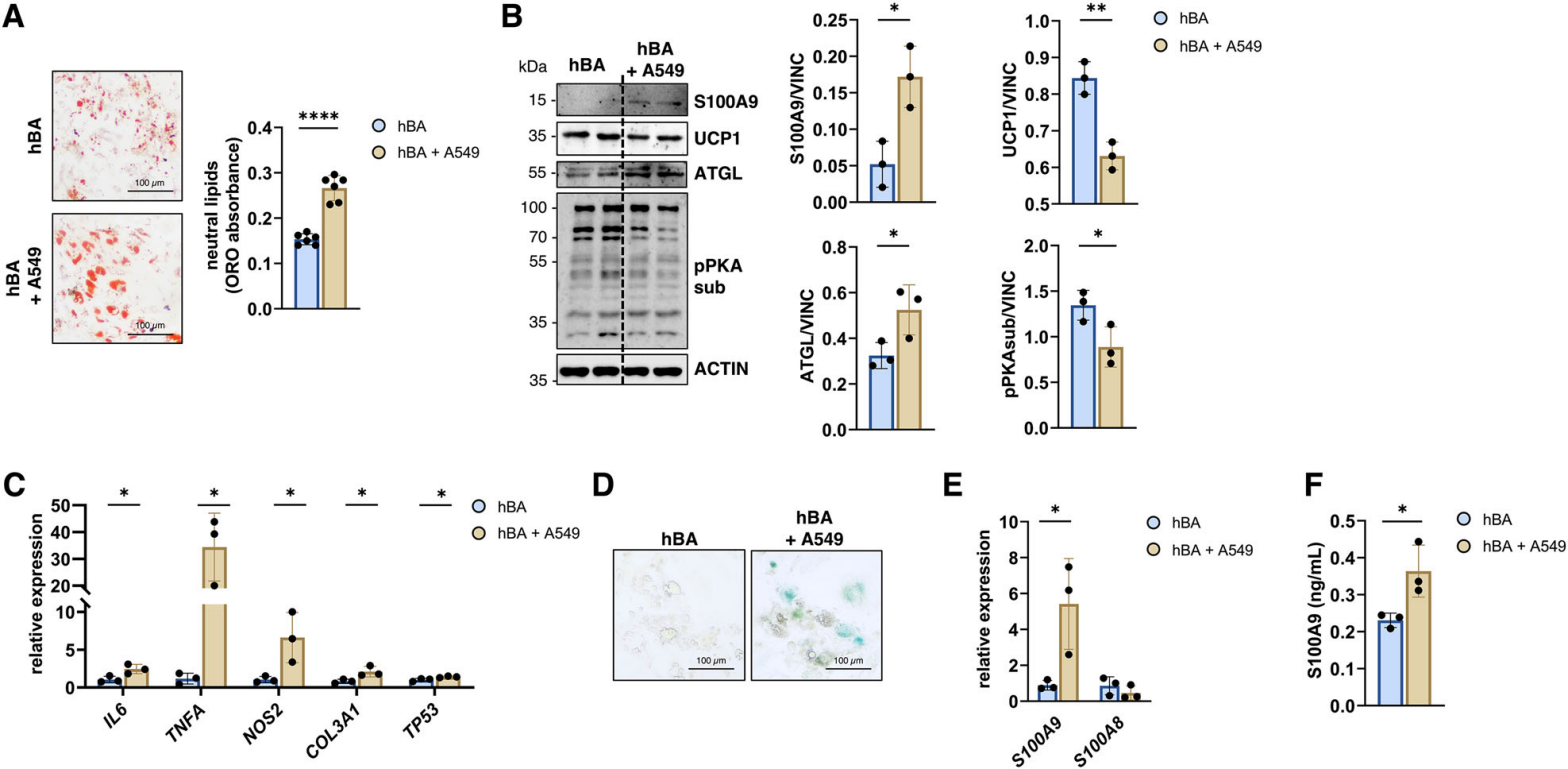
Human brown adipocytes recapitulate functional and inflammatory alterations in response to lung tumor-derived signals. **A** Oil Red O staining (*left panel*) and quantification of intracellular lipids (*right panel*) in immortalized human brown adipocytes (hBA) co-cultured with human A549 lung adenocarcinoma cells. Scale bar, 100 µm. Data are mean ± SD (*n* = 6; unpaired two-tailed *t*-test; ****p < 0.0001). **B** Representative immunoblot (*left panel*) and densitometry analysis (*right panel*) of S100A9, UCP1, ATGL, and pPKA sub levels in hBA brown adipocytes co-cultured with A549. β-actin (ACTIN) was used as loading control. Data are mean ± SD (*n* = 3; unpaired two-tailed *t*-test; *p < 0.05, **p < 0.01). **C** Relative mRNA expression of pro-inflammatory, fibrosis, and senescence markers (*IL6*, *TNFA*, *NOS2*, *COL3A1*, *TP53*) in hBA brown adipocytes following co-culture with A549 cells. Data are mean ± SD (*n* = 3; unpaired two-tailed *t*-test; *p < 0.05). **D** Representative SA-β-gal staining of hBA brown adipocytes after co-culture with A549 cells. Scale bar, 100 µm (*n* = 3). **E** Relative mRNA expression of *S100A9* and *S100A8* in hBA brown adipocytes following A549 co-culture. Data are mean ± SD (*n* = 3; unpaired two-tailed *t*-test; *p < 0.05). **F** ELISA quantification of S100A9 in culture supernatants from human brown adipocytes (hBA) under control conditions and after co-culture with A549 lung cancer cells, followed by removal of cancer cells and co-culture medium and recovery in fresh medium prior to supernatant collection. Data are presented as mean ± SD (*n* = 3 per group; unpaired two-tailed *t*-test; *p < 0.05).

To assess the translatability of our findings to human cells, we differentiated conditionally-immortalized human brown adipocytes (hBA) and exposed them to A549 lung adenocarcinoma cells. Upon co-culture, hBA exhibited increased accumulation of intracellular lipids, as revealed by Oil Red O staining (Fig. 6A), consistent with a lipid retention phenotype. Western blot analysis confirmed a significant elevation of S100A9 protein; moreover, downregulation of the thermogenic marker UCP1 and pPKA sub, along with increased levels of lipid droplet-associated ATGL, were observed, indicating impaired thermogenic and lipolytic function in human brown adipocytes co-cultured with A549 cells (Fig. 6B). In parallel, we observed a marked increase in *IL6*, *NOS2*, and *TNFA* mRNA expression (Fig. 6C), confirming activation of a pro-inflammatory program in response to tumor-derived signals. Consistently, hBA exposed to A549 cells developed a senescent phenotype, as shown by elevated mRNA levels of *COL3A1* and *TP53*, and enhanced SA-β-gal staining (Fig. 6C, D), recapitulating the transcriptional reprogramming observed in BAT and murine cultured brown adipocytes. Notably, S100A9 mRNA levels were robustly upregulated in hBA that were co-cultured with A549 cells (Fig. 6E), and S100A9 was efficiently released in *recovery* media collected after removal of A549 cells as assessed by ELISA (Fig. 6F). By contrast, S100A8 did not change upon A549 exposure (Fig. 6E), suggesting that hBA respond to lung tumor cues by enhancing S100A9 expression and potentially contributing to cachexia via this pro-inflammatory alarmin.

These findings demonstrate that human brown adipocytes undergo senescence, inflammatory activation, and thermogenic impairment when exposed to tumor-derived cues, suggesting a conserved mechanism of cancer-induced BAT dysfunction across species.

### Pro-cachectic and anti-thermogenic role of adipocyte-derived S100A9

**Fig. 7:**
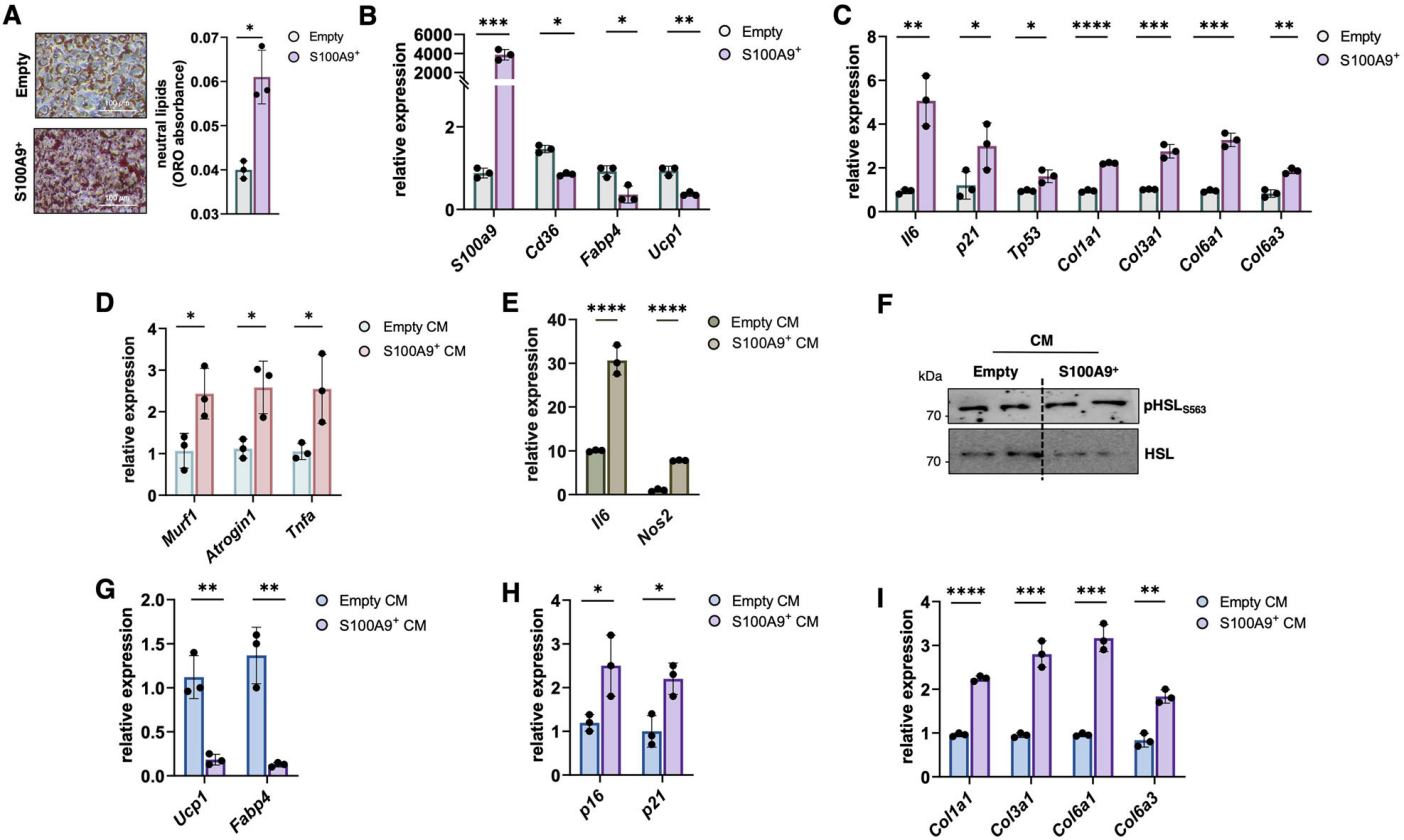
Brown adipocyte-derived S100A9 recapitulates cachexia-associated remodeling in brown and white adipocytes and promotes myotubes atrophy. **A** Oil Red O staining (*left panel*) and quantification of lipid content (*right panel*) in T37i brown adipocytes transfected with control plasmid (Empty) or S100A9-expressing construct (S100A9^+^). Scale bars, 100 µm. Data are mean ± SD (*n* = 3; unpaired two-tailed *t*-test; *p < 0.05). **B** Relative mRNA expression of *S100a9*, thermogenic and lipid metabolism genes (*Cd36*, *Fabp4, Ucp1*) in S100A9^+^ vs Empty adipocytes. Data are mean ± SD (*n* = 3; unpaired two-tailed *t*-test; *p < 0.05, **p < 0.01, ***p < 0.001). **C** Relative mRNA expression of inflammatory (*Il6*), senescence (*p21*, *Tp53*), and fibrotic markers (*Col1a1*, *Col3a1*, *Col6a1*, *Col6a3*) in S100A9^+^vs Empty adipocytes. Data are mean ± SD (*n* = 3; unpaired two-tailed *t*-test; *p < 0.05, **p < 0.01, ***p < 0.001, ****p < 0.0001). **D** Relative mRNA expression of atrophy-related genes (*Murf1*, *Atrogin1*) and *Tnfa* in C2C12 myotubes treated with conditioned media (CM) from Empty or S100A9^+^ adipocytes. Data are mean ± SD (*n* = 3; unpaired two-tailed *t*-test; *p < 0.05). **E** Relative mRNA expression of *Il6* and *Nos2* in 3T3-L1 white adipocytes treated with CM from S100A9^+^ or Empty adipocytes. Data are mean ± SD (*n* = 3; unpaired two-tailed *t*-test; ****p < 0.0001). **F** Representative immunoblot of basal and phospho-HSL (S563) in 3T3-L1 white adipocytes treated with CM from S100A9^+^ or Empty adipocytes. **G**–**I** Relative mRNA expression of *Ucp1*, *Fabp4, p16*, *p21, Col1a1*, *Col3a1*, *Col6a1, Col6a3* in T37i brown adipocytes exposed to CM from S100A9^+^ or Empty adipocytes. Data are mean ± SD (*n* = 3; unpaired two-tailed *t*-test; *p < 0.05, **p < 0.01, ***p < 0.001, ****p < 0.0001).

As we discovered that S100A9 originates from BAT adipocytes, we overexpressed it in cultured brown adipocytes to mimic its LLC-mediated increase. S100A9 overexpressing adipocytes (S100A9^+^) showed lipid accumulation compared to control (Empty) and recapitulated all the changes observed in CAC BAT, such as downregulation of the expression of *Ucp1* and other thermogenic genes (*Cd36*, *Fabp4*) as well as upregulation of SASP and fibrotic markers (*Il6*, *p21*, *Tp53*, collagen mRNAs) (Fig. 7A–C).

We then used conditioned media (CM) of S100A9^+^ adipocytes to recapitulate some of the analyses performed on myocytes, white, and brown adipocytes treated with CAC BAT secretome. Of note, atrophy (*Murf1*, *Atrogin1*) and inflammatory marker *Tnfa* were induced in myotubes (Fig. 7D). Inflammatory gene expression (*Il6*, *Nos2*) and lipolysis (increased phospho-active HSL) were promoted in white adipocytes (Fig. 7E, F). On brown adipocytes, CM of S100A9^+^ adipocytes was able to promote downregulation of thermogenic (*Ucp1*, *Fabp4*) and upregulation of SASP genes (*p16*, *p21*, collagens) (Fig. 7G–I).

**Fig. 8:**
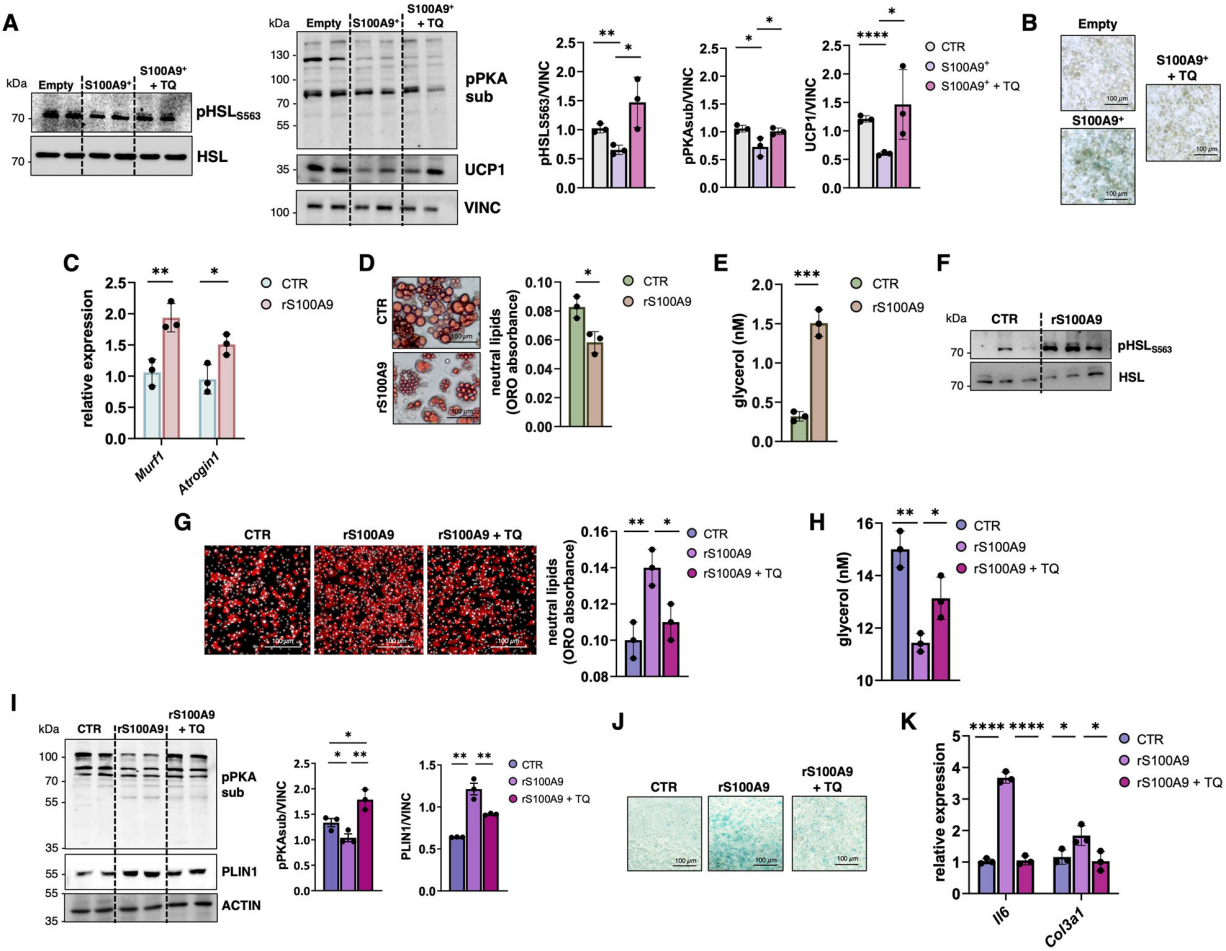
Recombinant S100A9 induces cachectic-like changes in white and brown adipocytes that are counteracted by Tasquinimod. **A** Representative immunoblot (*left panel*) and quantification (*right panel*) of basal and phospho-HSL (pHSL_S563_), PKA phospho-substrates (pPKA sub), and UCP1 in T37i brown adipocytes transfected with control plasmid (Empty) or S100A9-expressing construct (S100A9^+^) ± Tasquinimod (TQ). Data are mean ± SD (*n* = 3; one-way ANOVA with Tukey’s post hoc; *p < 0.05, **p < 0.01, ****p < 0.0001). **B** Representative SA-β-gal staining of Empty and S100A9^+^ adipocytes ± TQ. Scale bar, 100 µm (*n* = 3). **C** Relative mRNA expression of atrophy-related genes (*Murf1*, *Atrogin1*) in C2C12 myotubes treated with vehicle (CTR) or recombinant S100A9 (rS100A9) protein. Data are mean ± SD (*n* = 3; unpaired two-tailed *t-*test; *p < 0.05, **p < 0.01). **D** Oil Red O staining (*left panel*) and quantification of intracellular lipid content (*right panel*) in 3T3-L1 white adipocytes treated with rS100A9 protein. Scale bar, 100 µm. Data are mean ± SD (*n* = 3; unpaired two-tailed *t*-test; *p < 0.05). **E** Quantification of glycerol release in culture media from 3T3-L1 white adipocytes following rS100A9 treatment. Data are mean ± SD (*n* = 3; unpaired two-tailed *t*-test; ***p < 0.001). **F** Representative immunoblot of phospho-HSL (pHSL_S563_) in 3T3-L1 white adipocytes treated with rS100A9. **G** Representative immunofluorescence images (*left panel*) and neutral lipid quantification (*right panel*) of Oil Red O stained T37i adipocytes treated with rS100A9 ± TQ. Data are mean ± SD (*n* = 3; one-way ANOVA with Tukey’s post hoc; *p < 0.05, **p < 0.01). **H** Quantification of glycerol release in culture media from T37i brown adipocytes treated with rS100A9 ± TQ. Data mean ± SD (*n* = 3; one-way ANOVA with Tukey’s post hoc; *p < 0.05, **p < 0.01). **I** Representative immunoblot (*left panel*) and quantification (*right panel*) of PKA phospho-substrates (pPKA sub) and PLIN1 in T37i brown adipocytes treated with rS100A9 ± TQ. β-actin (ACTIN) was used as loading control. Data mean ± SD (*n* = 3; one-way ANOVA with Tukey’s post hoc; *p < 0.05, **p < 0.01). **J** Representative β-galactosidase staining in T37i brown adipocytes treated with rS100A9 ± TQ. Scale bar, 100 µm (*n* = 3). **K** Relative mRNA expression of *Il6* and *Col3a1* in T37i brown adipocytes treated with rS100A9 ± TQ. Data are mean ± SD (*n* = 3; one-way ANOVA with Tukey’s post hoc; *p < 0.05, ****p < 0.0001).

We treated S100A9^+^ adipocytes with Tasquinimod (TQ), a small molecule that binds S100A9 and interferes with its interaction with both TLR4 and RAGE receptors [45] to directly assess the involvement of S100A9/TLR4-RAGE pathway in the observed detrimental effects. The atrophic and lipolytic effects of S100A9 have been previously reported [25, 46], while its anti-thermogenic effects have not been investigated yet. Hence, prior to TQ treatment, we firstly checked and confirmed the *Rage* and *Tlr4* expression in brown adipocytes (Fig. S3A). TQ treatment restored physiological lipolytic activity in brown adipocytes, as evidenced by increased levels of the phospho-active form of HSL (pHSL_S563_) and of the overall levels of pPKA substrates (Fig. 8A). Moreover, TQ treatment reinstated the thermogenic capacity of brown adipocytes, indicated by the accumulation of UCP1 (Fig. 8A). Notably, TQ also prevented the acquirement of the SA-β-gal positivity (Fig. 8B).

To further investigate the molecular function of S100A9, we next employed treatments with its purified recombinant form (rS100A9).

As expected, mRNA levels of atrophy genes (*Murf1* and *Atrogin1*) were found increased in myotubes when treated with rS100A9 protein compared to CTR (Fig. 8C) and accompanied by upregulation of mRNA levels of *Il6, Tnfa,* and *Nos2* (Fig. S3B). In white adipocytes, rS100A9 promoted the upregulation of inflammatory genes (*Il6*, *Nos2, Tnfa*), and this was correlated to activation of the main kinases governing immune responses, such as AMPK, NFkB, and p38 (Fig. S3C, D). Notably, rS100A9 treatment evoked intracellular lipid reduction in white adipocytes (Fig. 8D), consistent with induced lipolysis as demonstrated by increased level of glycerol release and phospho-active HSL compared to untreated white adipocytes (Fig. 8E, F).

Treatment with rS100A9 alone successfully recapitulated major features of cachectic BAT ex vivo (Fig. S4A–C). In vitro, rS100A9 treatment reduced brown adipocyte lipolysis (Fig. 8G–I), elevated SA-β-gal activity (Fig. 8J), and increased inflammatory and collagen genes (Fig. 8K). TQ treatment effectively counteracted the detrimental effects of rS100A9 on brown adipocytes. Actually, co-treatment with TQ led to a reduction in intracellular lipid accumulation and restored the phosphorylation of PKA substrates, suggesting a significant recovery of brown adipocyte identity (Fig. 8G–I). In parallel, TQ significantly reduced SA-β-gal staining, indicating prevention of cellular senescence (Fig. 8J). Gene expression analysis confirmed that TQ attenuated the rS100A9-induced upregulation of key inflammatory (*Il6*) and collagen (*Col3a1*) genes (Fig. 8K).

Altogether, these findings demonstrate that pharmacological blockade of S100A9 signaling is sufficient to revert the key molecular and functional features of brown adipocyte dysfunction, supporting a direct role for S100A9 in driving BAT remodeling during CAC.

## Discussion

CAC profoundly affects adipose tissues, yet the contribution of BAT to this syndrome remains debated. In mice, tumor-derived cues can induce browning of subcutaneous white adipose tissue (sWAT) and hyperactivation of thermogenic pathways, thereby increasing energy expenditure and aggravating tissue wasting [12, 13]. These findings established the prevailing view that BAT activation—or sWAT browning—exacerbates the energetic imbalance driving cachexia.

However, this paradigm has been challenged by emerging clinical data in humans. Retrospective ^FDG^PET/CT studies have shown no consistent association between BAT activity and cancer-related weight loss [16], and recent longitudinal analyses suggest that detectable BAT at diagnosis may correlate with attenuated weight loss over time [17]. Moreover, a prospective study in breast cancer patients found that BAT activation was associated with longer progression-free survival, supporting the notion that metabolically active BAT may exert a protective effect in certain oncologic settings [47]. Although increased BAT activity has been observed in selected tumors, such as breast cancer [48]. Its detectability declines in older or advanced-stage patients [47]—and neither presence nor absence of FDG uptake reliably predicts cachexia severity. This discrepancy underscores the need to move beyond the simplistic notion of BAT as an energy-dissipating tissue and to investigate how BAT is qualitatively remodeled in response to tumor burden. Indeed, mounting clinical and experimental evidence suggests that BAT during ageing, and upon different stress conditions, may undergo a functional transition, losing its thermogenic capacity and acquiring a pro-inflammatory and dysfunctional phenotype [49–53].

Our study provides compelling evidence that chronic tumor-derived stress induces a senescence-like conversion of BAT, leading to loss of oxidative capacity and acquisition of a secretory phenotype reminiscent of the SASP. This is characterized by upregulation of pro-inflammatory cytokines, collagen production, and senescence markers. Notably, IL-6 was among the cytokines upregulated in cachectic BAT. IL-6 is a pleiotropic mediator with context-dependent effects on adipose tissue, as it has been implicated both in thermogenic activation and browning responses as well as in the suppression of BAT thermogenic competence [54, 55]. In our CAC setting, where BAT undergoes remodeling and senescence-like conversion, IL-6 is likely part of a broader SASP-like inflammatory program [56], thereby contributing to BAT dysfunction and thermogenic inactivation. These results are in line with observations in sWAT of cachectic patients, who show increased fibrosis and enhanced production of collagens and inflammatory cytokines [57, 58]. However, in sWAT this is accompanied by immune cell infiltration, which appears to drive fibrosis. In contrast, in our model of cachectic BAT, brown adipocytes themselves—not immune cells—are the principal source of this SASP-like response. Our data indicate that brown adipocytes can autonomously respond to tumor-induced cues by activating a pathological secretory program.

This senescence-associated shift in BAT identity may be particularly relevant to humans, whose BAT is intrinsically less thermogenically potent than that of rodents and more susceptible to inflammatory and metabolic stress [49, 50]. Although functional UCP1 is present in human brown adipocytes, the total BAT thermogenic capacity in adults is significantly lower due to reduced BAT mass and mitochondrial density. Age, obesity, and chronic systemic inflammation—common features in cancer patients—predispose adipose tissue to dysfunction and senescence [59–61]. Under these conditions, BAT may shift from a metabolically protective tissue to a driver of systemic deterioration by releasing deleterious inflammatory mediators.

A pivotal finding of our study is the identification of S100A9 as a major BAT-derived factor within this altered secretome. S100A9 has previously been implicated in muscle wasting and adipose tissue dysfunction in obesity [62–64]. Our data demonstrate for the first time that brown adipocytes themselves produce and secrete S100A9 in response to cancer exposure, independently of infiltrating immune cells. Notably, S100A9 levels rise more significantly than S100A8 in cachectic BAT, and this is confirmed in human brown adipocytes stimulated with lung cancer-derived factors, suggesting an autonomous function for S100A9 beyond the classical S100A8/A9 heterodimer. This is consistent with prior reports indicating that S100A9 homodimers can activate TLR4 and RAGE signaling pathways independently of S100A8 [65, 66].

Functionally, S100A9 protein alone is sufficient to reproduce key features of cachectic BAT remodeling—including impaired thermogenic gene expression, fibrosis, inflammation, and senescence—thus establishing it as a central effector of BAT pathological state. These effects were both autocrine (on brown adipocytes) and paracrine, promoting lipolysis in white adipocytes and atrophy in skeletal myotubes. This expands the known repertoire of BAT-secreted factors (“batokines”), positioning S100A9 as a novel and deleterious component of the cachectic “signalome”.

A key mechanistic aspect emerging from our data is that S100A9 elicits divergent metabolic outputs in white and brown adipocytes despite activating the same innate immune signaling axis. While in white adipocytes S100A9 promoted activation of lipolysis, in brown adipocytes it suppressed the PKA-dependent program required for coordinated lipolysis and thermogenesis. Notably, the inhibitory effect of S100A9 on the thermogenic program was directly observed in differentiated brown adipocytes in vitro, supporting a cell-autonomous mechanism. A plausible explanation is that inflammatory signaling downstream of TLR4 can sustain catabolic lipid mobilization in white adipocytes, consistent with the established contribution of inflammation-driven lipolysis to CAC [67]. By contrast, brown adipocytes critically depend on tight coupling between PKA-driven lipolysis and mitochondrial oxidative capacity to maintain UCP1-dependent thermogenic competence, and TLR4 activation has been shown to antagonize adaptive thermogenesis, suppressing UCP1 induction and impairing thermogenic competence, in association with ER stress and mitochondrial dysfunction [68]. Thus, although cachexia can promote WAT remodeling characterized by induction of beige-like markers, our data suggest that S100A9-driven inflammatory signaling may simultaneously amplify catabolic lipid mobilization in white adipocytes while suppressing the functional thermogenic program in classical brown adipocytes.

Our findings are further corroborated by Wang et al. [69], who identified S100A8/A9 as key factors secreted by brown adipocytes in a model of Rheb-deficient BAT, where genetic mTOR suppression led to whitening, loss of UCP1, lipid accumulation, and impaired thermogenesis. Like in our study, secretion of S100A8/A9 occurred independently of immune infiltration. However, the authors did not dissect the individual contribution of S100A9 nor its wasting effects. Importantly, their findings of BAT whitening and alarmin secretion align with our model, further supporting the concept of BAT reprogramming into a secretory organ.

Our study extends these observations to the pathophysiological setting of CAC, showing that tumor-derived cues—not artificial genetic manipulations—drive BAT whitening and dysfunction. The more pronounced upregulation of S100A9, validated in both murine and human brown adipocytes, underscores its selective and pathogenic role. Furthermore, we show that S100A9 is sufficient to trigger hallmark features of cachectic BAT, and its effects extend beyond BAT to white adipocytes and skeletal muscle cells, implying it as a potent endocrine mediator.

Taken together, these findings converge on a shared conceptual framework in which dysfunctional BAT, undergoing whitening, transitions from a thermogenic to a secretory organ, releasing inflammatory alarmins such as S100A9 that can propagate systemic tissue damage. Our work expands this paradigm by identifying S100A9 not only as a biomarker but also as a causal driver of BAT senescence and cachexia progression. Additionally, in pancreatic cancer-associated cachexia, circulating S100A9 levels correlate with muscle atrophy and weight loss in patients, and experimental S100A9 exposure induces myotube atrophy in vitro [25].

Mechanistically, we confirmed the role of the S100A9–TLR4/RAGE axis in BAT inflammation and senescence by pharmacologically targeting this pathway with Tasquinimod. This treatment reversed S100A9-induced molecular changes, restoring thermogenic gene expression and attenuating inflammatory and senescence markers. While TLR4 signaling has been previously implicated in muscle and WAT wasting during cachexia [70], our findings extend its relevance to BAT, supporting its inclusion as a critical node in the systemic cachexia network. Nonetheless, Tasquinimod served explicitly as a proof-of-concept to validate the molecular involvement of the S100A9 signaling rather than as direct evidence for clinical utility. Therefore, dedicated validation studies are still needed.

Despite the comprehensive mechanistic framework provided by our findings, several intrinsic limitations warrant discussion. First, although our study characterizes a novel secretory and senescent phenotype in BAT during CAC, the precise initiating molecular signals and temporal dynamics orchestrating this phenotypic transition remain incompletely understood and require future targeted investigations. Furthermore, while we clearly demonstrate the autocrine function of S100A9, the complete spectrum of SASP-related secreted factors involved in BAT-mediated pro-cachectic effects was not fully explored. Future research employing unbiased proteomic and secretomic profiling approaches could identify additional key mediators contributing to BAT-driven cachexia.

In conclusion, BAT in cancer cachexia is not merely dysfunctional—it is actively reprogrammed into a pro-inflammatory, senescent, white-like organ that contributes to systemic wasting via factors such as S100A9. Preserving BAT’s integrity and preventing its senescent transition may be beneficial, whereas strategies that indiscriminately promote BAT stimulation could be ineffective or deleterious in this context. From a translational standpoint, our findings help explain the heterogeneity observed in clinical studies of BAT in cancer and underscore the need for biomarkers that reflect BAT’s functional state. Ultimately, decoding the BAT-derived secretome may unveil new therapeutic and diagnostic strategies in cancer cachexia.

## Supplementary information

Supplementary figures

Original Western Blots

Proteomics data

## Data Availability

RNA-seq data have been deposited in the GEO database under accession number GSE304220. Proteomics data are available as supplementary material. All other data supporting the findings of this study are available from the lead contact upon reasonable request. Any additional information necessary to reanalyze the data reported in this paper is also available from the lead author upon reasonable request. Source data are provided with this paper.
