# Supplementary material for: Cancer cachexia induces senescent reprogramming of brown adipose tissue and pro-cachectic S100A9 secretion by adipocytes
Source: Cell Death Dis. 2026 May 2;17(1):584. doi: 10.1038/s41419-026-08806-x (PMC13280486; doi:10.1038/s41419-026-08806-x)
Supplement: Supplementary file 1 — Supplementary figures [file 41419_2026_8806_MOESM1_ESM.pdf]

Cancer cachexia induces senescent reprogramming of brown adipose tissue and pro-cachectic S100A9 secretion by adipocytes

Claudia Di Biagio, Flavia Tortolici, Francesco Gaudioso, Andrea Ninni, Francesca Giurdanella Annina, Chiara De Ranieri, Fabio Zaccaria, Francesca Sciarretta, Luca Verteramo, Francesca Arciprete, Simone Carotti, Antoine AF de Vries, Sander Kooijman, Francesca Pacello, Andrea Battistoni, Daniele Lettieri-Barbato, Katia Aquilano

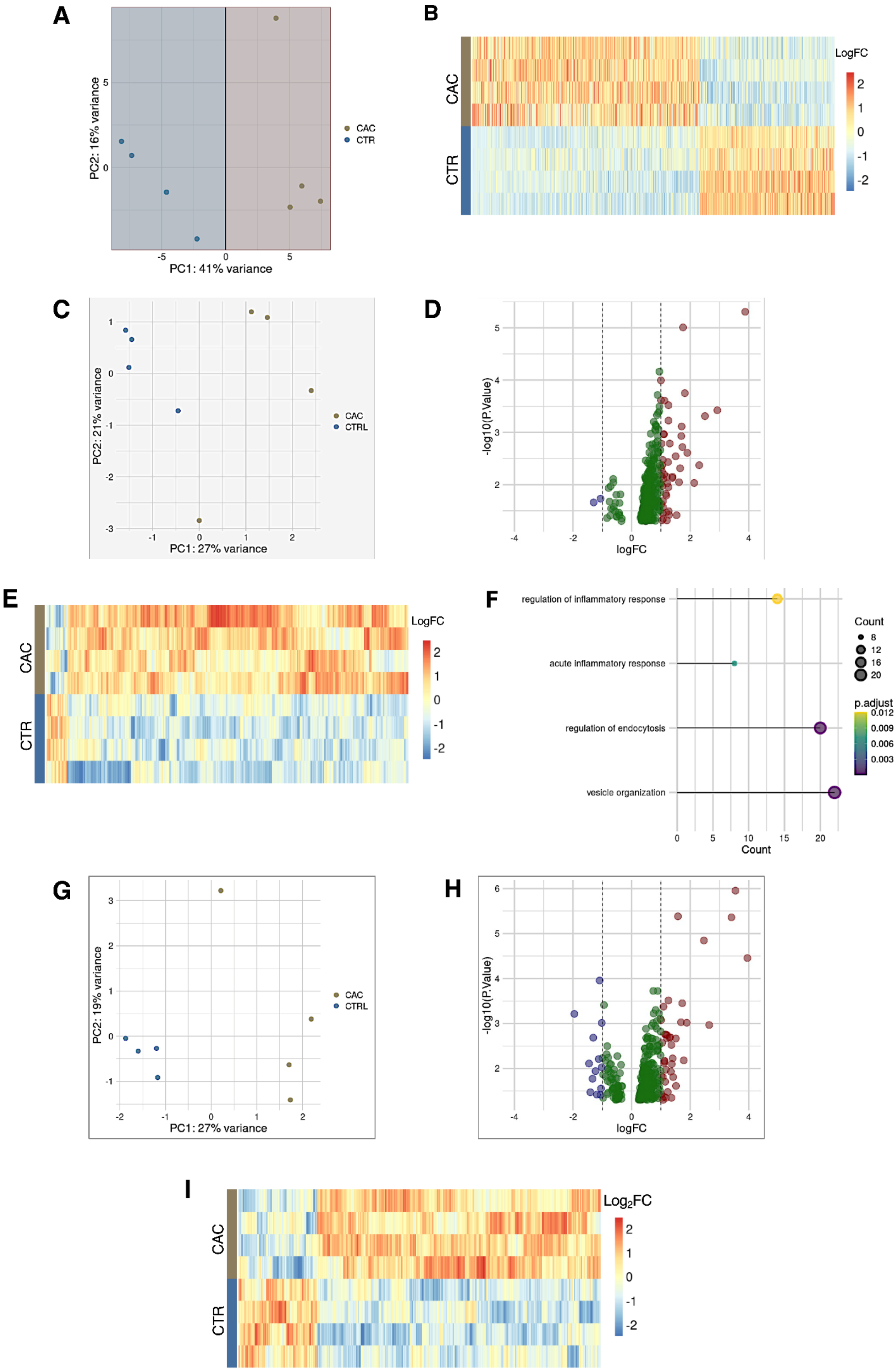
A
PC2: 16% variance
PC1: 41% variance
CAC
CTR
B
CAC
CTR
LogFC
C
PC2: 21% variance
PC1: 27% variance
CAC
CTRL
D
-log10(P.Value)
logFC
E
CAC
CTR
LogFC
F
regulation of inflammatory response
acute inflammatory response
regulation of endocytosis
vesicle organization
Count
p.adjust
G
PC2: 19% variance
PC1: 27% variance
CAC
CTRL
H
-log10(P.Value)
logFC
I
CAC
CTR
Log2FC

Supplementary Fig. 1 | Transcriptomic and proteomic profiling of BAT reveals extensive inflammatory and metabolic reprogramming in cancer cachexia.
A, Principal component analysis (PCA) of RNAseq data distinguishes BAT samples from CTR and LLC-bearing cachectic (CAC) mice (n = 4 per group).
B, Heatmap related to RNAseq of differentially expressed genes (DEGs) in BAT from CTR and CAC mice (n = 4; $p < 0.05$; log2FC < -0.5; log2FC >0.5).
C, PCA of proteomics data performed on BAT distinguishes samples from CAC and CTR mice (n = 4 per group).
D, Volcano plot of proteomic data on BAT samples showing distribution of upregulated (red), downregulated (blue), and non-significant (grey) protein from CAC vs CTR mice ($p < 0.05$).
E, Heatmap of differentially expressed proteins in BAT from CAC and CTR mice ($p < 0.05$; log2FC < -0.5; log2FC >0.5).
F, Gene Set Enrichment Analysis for Gene Ontology (GO) Biological Process of upregulated proteins from proteomics data in BAT of CAC vs CTR mice (p adj < 0.05; log2FC>0.5).
G, PCA of proteomic data performed on BAT secretome distinguishes BAT samples from CAC and CTR mice *(n* = 4 per group).
H, Volcano plot of proteomic data on BAT secretome showing distribution of upregulated (red), downregulated (blue), and non-significant (grey) protein from CAC vs CTR mice ($p < 0.05$).
I, Heatmap of differentially represented proteins in BAT secretome from CAC vs CTR mice ($p < 0.05$; log2FC < -0.5; log2FC >0.5).

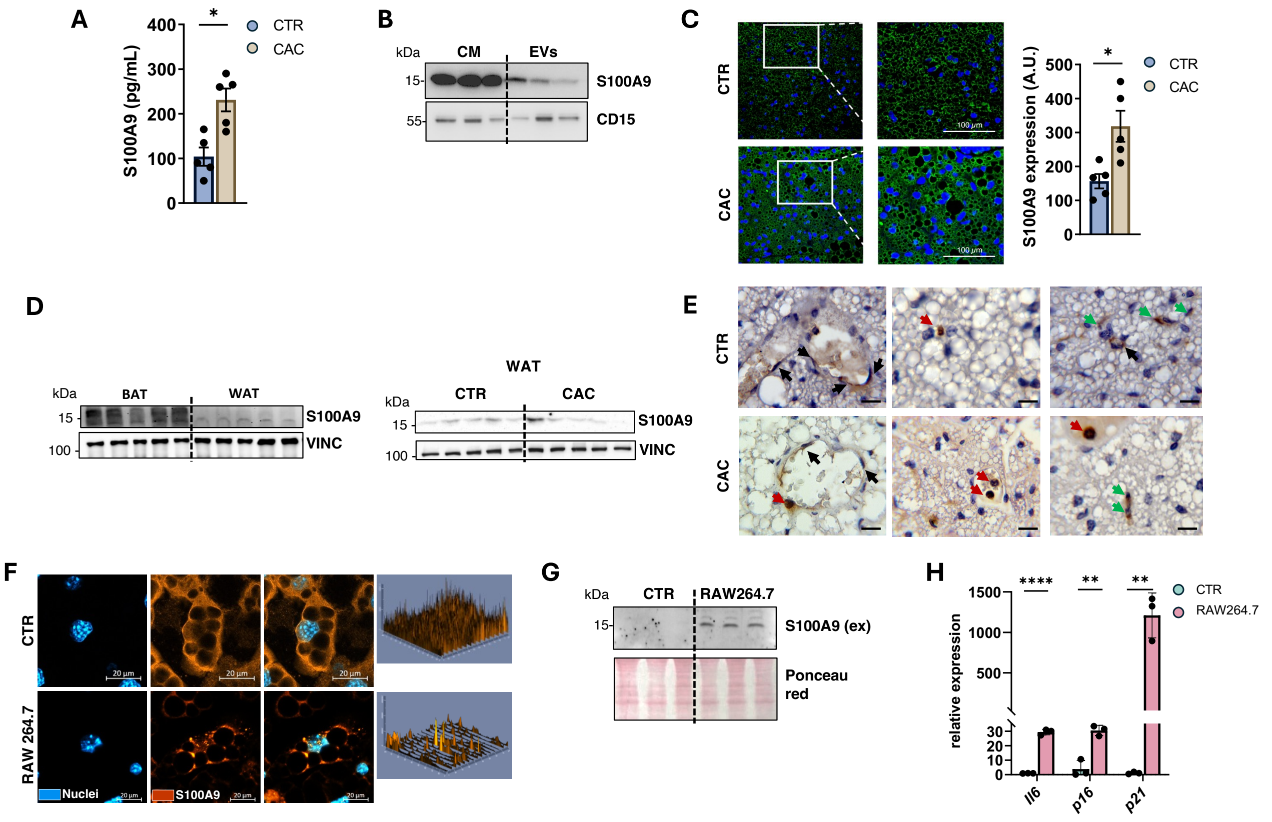

Supplementary Fig. 2 | Increased S100A9 release from brown adipocytes and induction of a senescent-inflammatory phenotype upon macrophage co-culture.

A, Quantification of S100A9 in plasma from control (CTR) and cachectic (CAC) mice. Data are mean ± SD (n = 5 per group; unpaired two-tailed t-test; *p < 0.05).

B, Immunoblot of S100A9 in BAT conditioned medium (CM) and isolated BAT extracellular vesicles (EVs) of CTR and CAC mice. CD15 was used as loading control (n = 3).

C, Representative immunofluorescence images (*left panel*) and quantification (*right panel*) of S100A9 (green) in BAT sections from CTR and CAC mice. Nuclei are stained with Hoechst 33342 (blue). Scale bars, 100 µm. Data are mean ±SD (n = 5; unpaired two-tailed t-test; *p < 0.05).

D, Comparison of basal S100A9 protein between BAT and visceral WAT (*left panel*) and S100A9 protein levels in visceral WAT of CAC mice (*right panel*). VINCULIN (VINC) was used as loading control (n = 5 for each group).

E, S100A9 expression identified by morphological criteria in BAT from CTR and CAC mice within endothelial cells, neutrophils, and fibroblasts as indicated by black, red, and green arrows, respectively). Scale bars, 20 µm (100× magnification).

F, Representative immunofluorescence images stained for S100A9 (red) in mBA brown adipocytes co-cultured with control or LPS-stimulated RAW macrophages. Nuclei are stained with Hoechst 33342 (blue). Right panels show 3D surface plots of fluorescence intensity distribution, representing intracellular localization and abundance of S100A9. Scale bars, 20 µm.

G, Immunoblot of S100A9 protein in conditioned media (ex) of mBA brown adipocytes after recovery of co-culture treatment with LPS-activated RAW264.7 macrophages. Ponceau red staining was used as loading control.

H, Relative mRNA expression of *Il6*, *p16*, and *p21* in mBA brown adipocytes after co-culture with LPS-stimulated RAW macrophages. Data are mean ± SD (n = 3; unpaired two-tailed t-test; **p < 0.01, ****p < 0.0001).

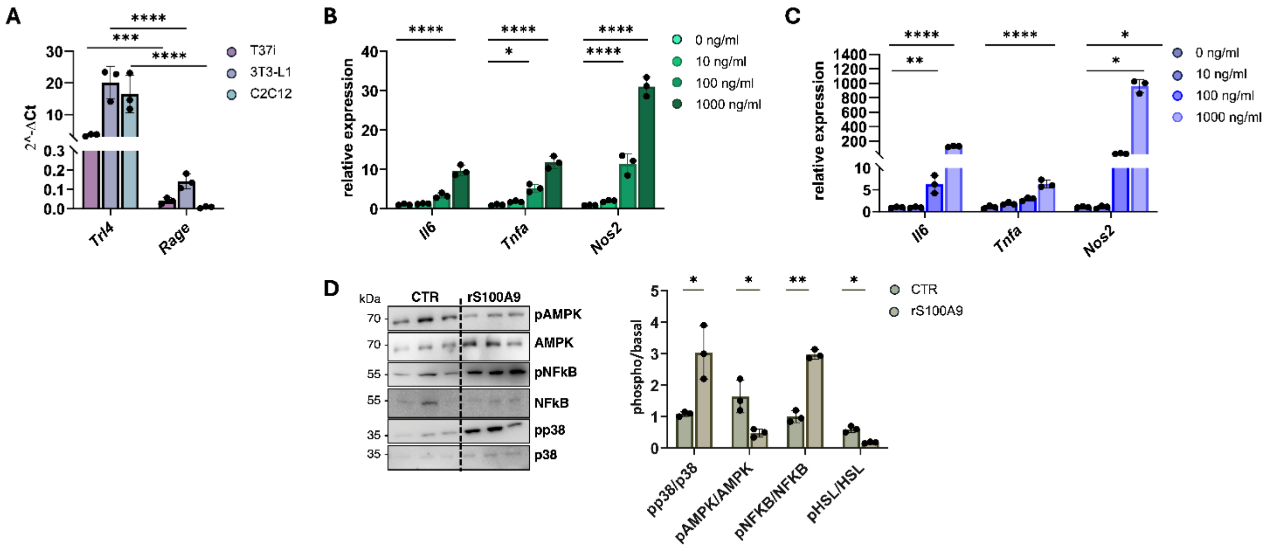

Supplementary Fig. 3 | Recombinant S100A9 induces inflammatory, atrophic and kinase activation programs in myotubes and adipocytes.

A, Relative mRNA expression of S100A9 receptors (*Tlr4*, *Rage*) in myotubes, brown and white adipocytes. Data are mean ± SD ($n = 3$; one-way ANOVA, Tukey's post hoc test; ***$p < 0.001$, ****$p < 0.0001$).

B, C, Relative mRNA expression of inflammatory genes (*Il6*, *Nos2* and *Tnfa*) in C2C12 myotubes (a) and 3T3-L1 white adipocytes (b) treated for 24 h with recombinant S100A9 (rS100A9) at different doses. Data are mean ± SD ($n = 3$; unpaired two-tailed t-test; *$p < 0.05$, **$p < 0.01$, ****$p<0.0001$).

D, Immunoblot (*left panel*) and quantification (*right panel*) of basal and phosphorylated p38, AMPK, and NF-κB, in 3T3-L1 white adipocytes treated with rS100A9. Quantifications are presented as phospho/basal protein ratio. Data are mean ± SD ($n = 3$; unpaired two-tailed t-test; *$p < 0.05$, **$p < 0.01$).

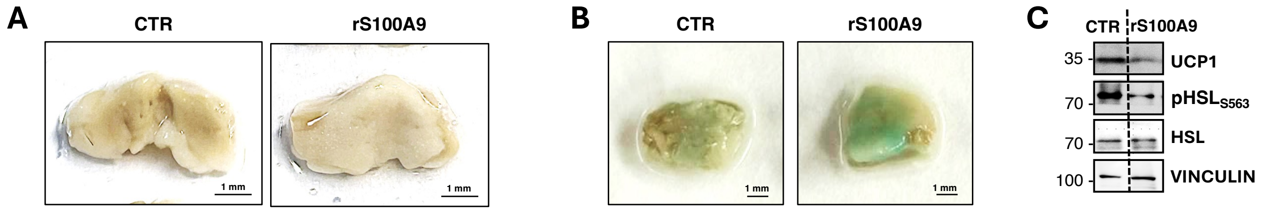

Supplementary Fig. 4 | *Ex vivo* rS100A9 injection induces whitening and a senescence-associated phenotype in BAT.

A, Representative photographs of whole BAT explants immediately injected *ex vivo* with PBS vehicle (CTR) or recombinant S100A9 (rS100A9) and subsequently maintained in culture overnight (n=3).

B, Senescence-associated β-galactosidase (SA-β-gal) staining of a representative BAT lobe following overnight culture after *ex vivo* injection with PBS or rS100A9 (n=3).

C, Representative immunoblot analysis of UCP1 and hormone-sensitive lipase (HSL), including total HSL and phosphorylated HSL at Ser563 ($pHSL_{S563}$), in BAT explants injected *ex vivo* with PBS or rS100A9 (*left panel*). Vinculin was used as loading control.
