# Supplementary material for: Cancer cachexia induces senescent reprogramming of brown adipose tissue and pro-cachectic S100A9 secretion by adipocytes
Source: Cell Death Dis. 2026 May 2;17(1):584. doi: 10.1038/s41419-026-08806-x (PMC13280486; doi:10.1038/s41419-026-08806-x)

FIGURE 2

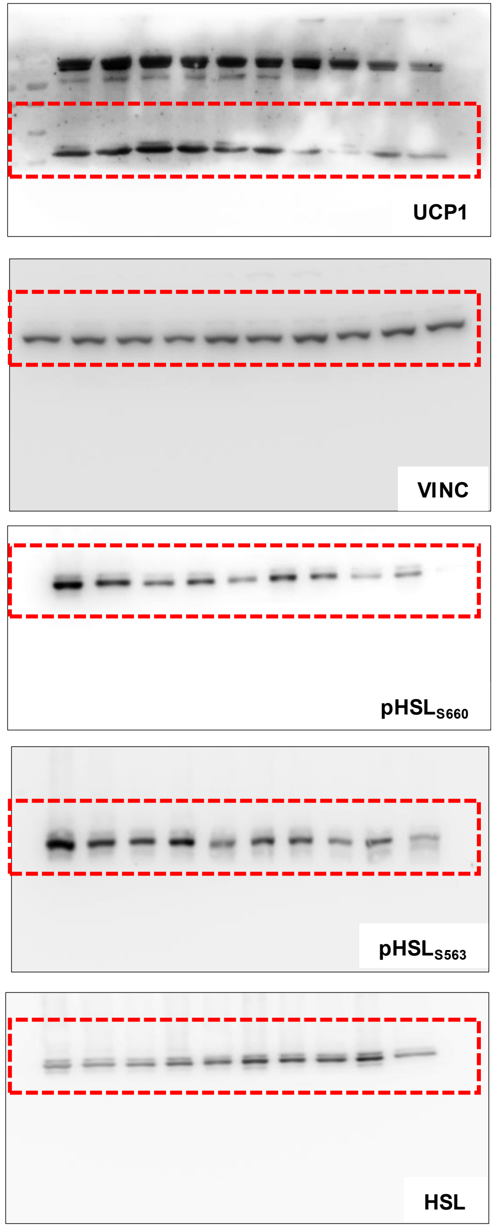

FIGURE 3

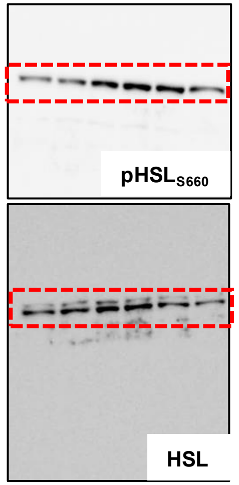

FIGURE 4

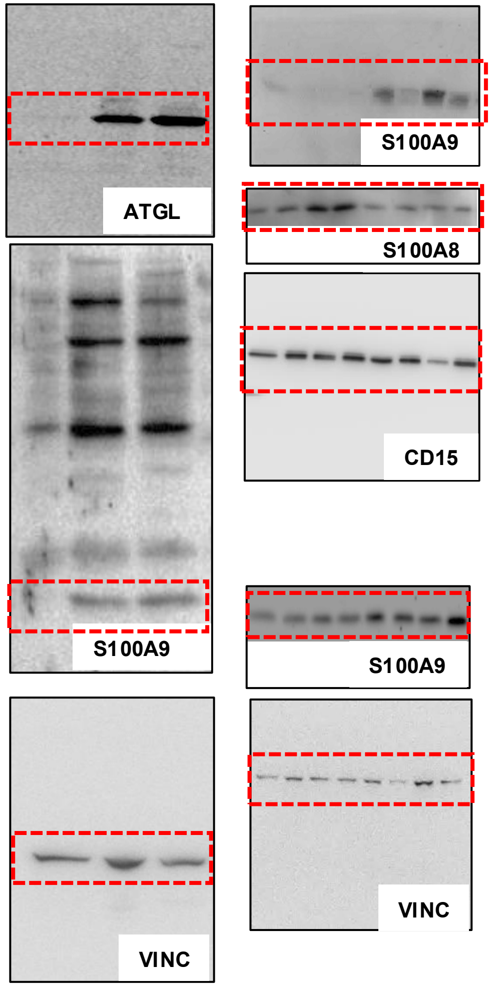

FIGURE 5

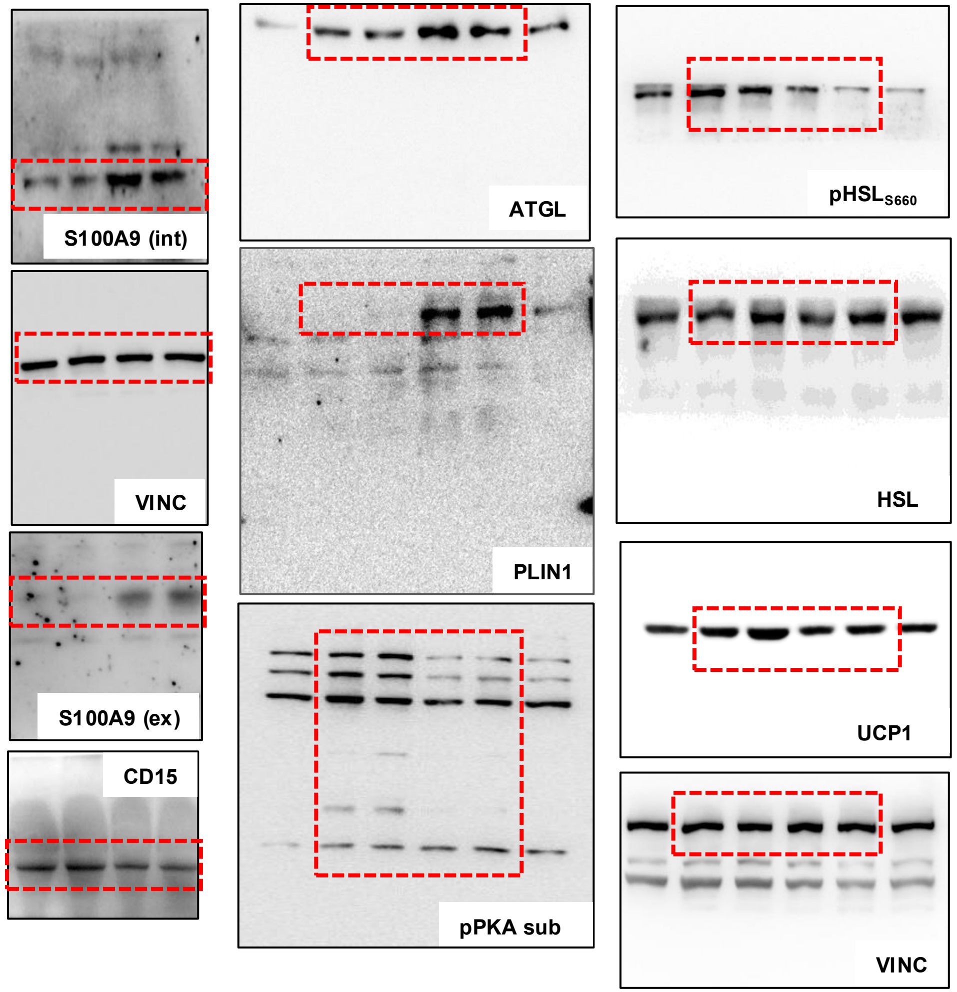

FIGURE 6

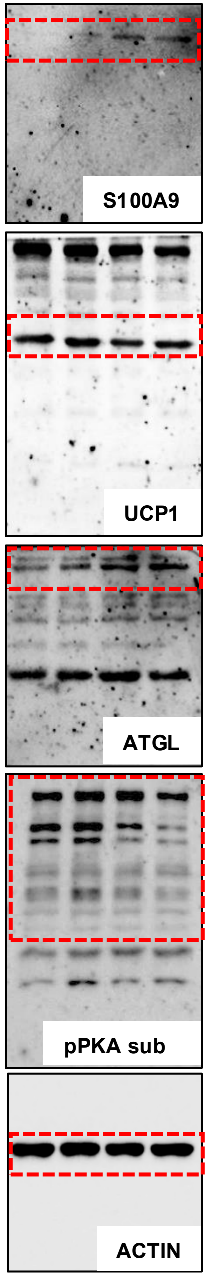

FIGURE 7

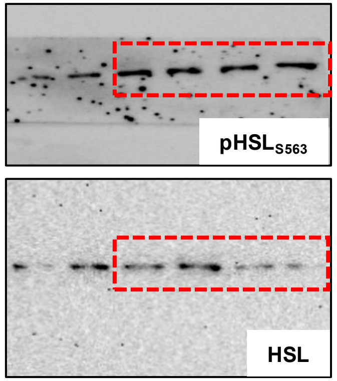

FIGURE 8

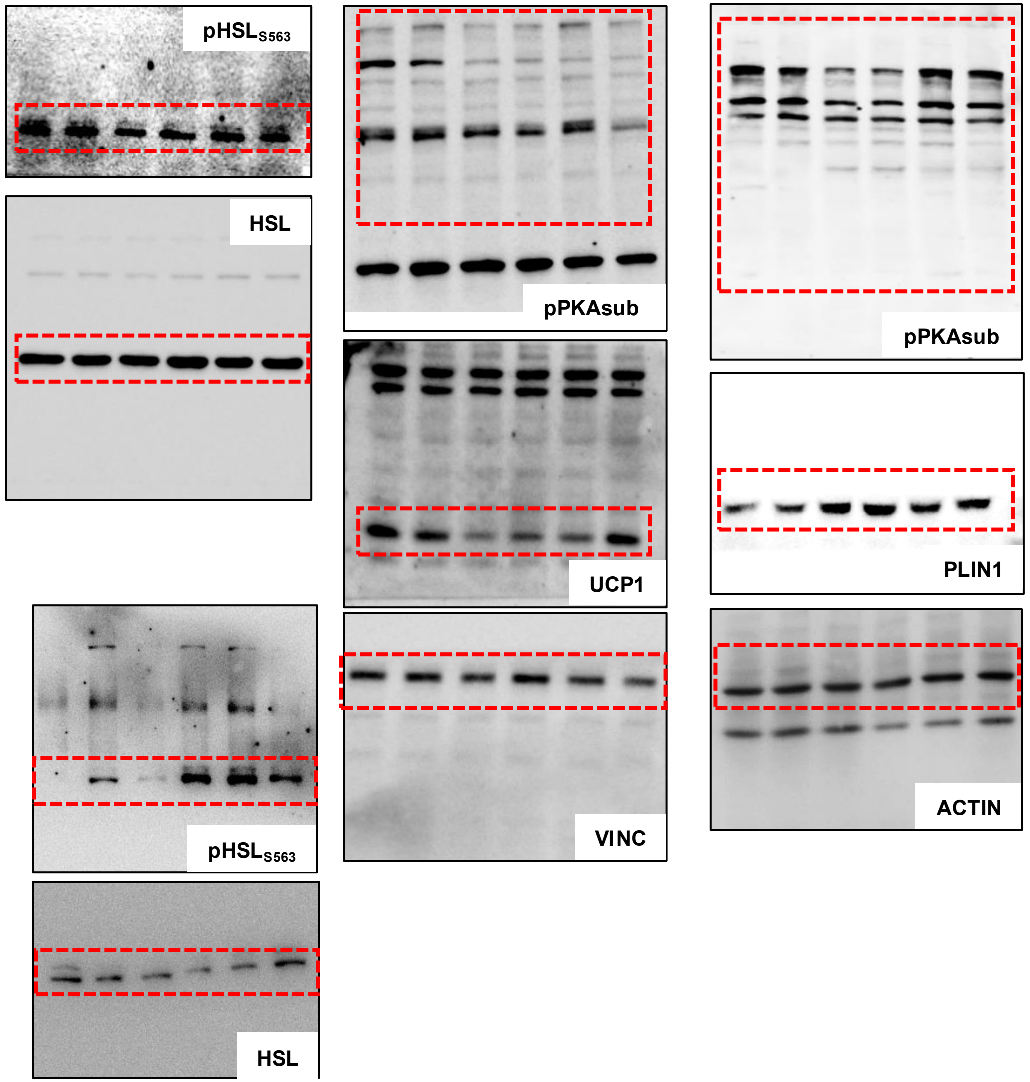

FIGURE 2
extended Data

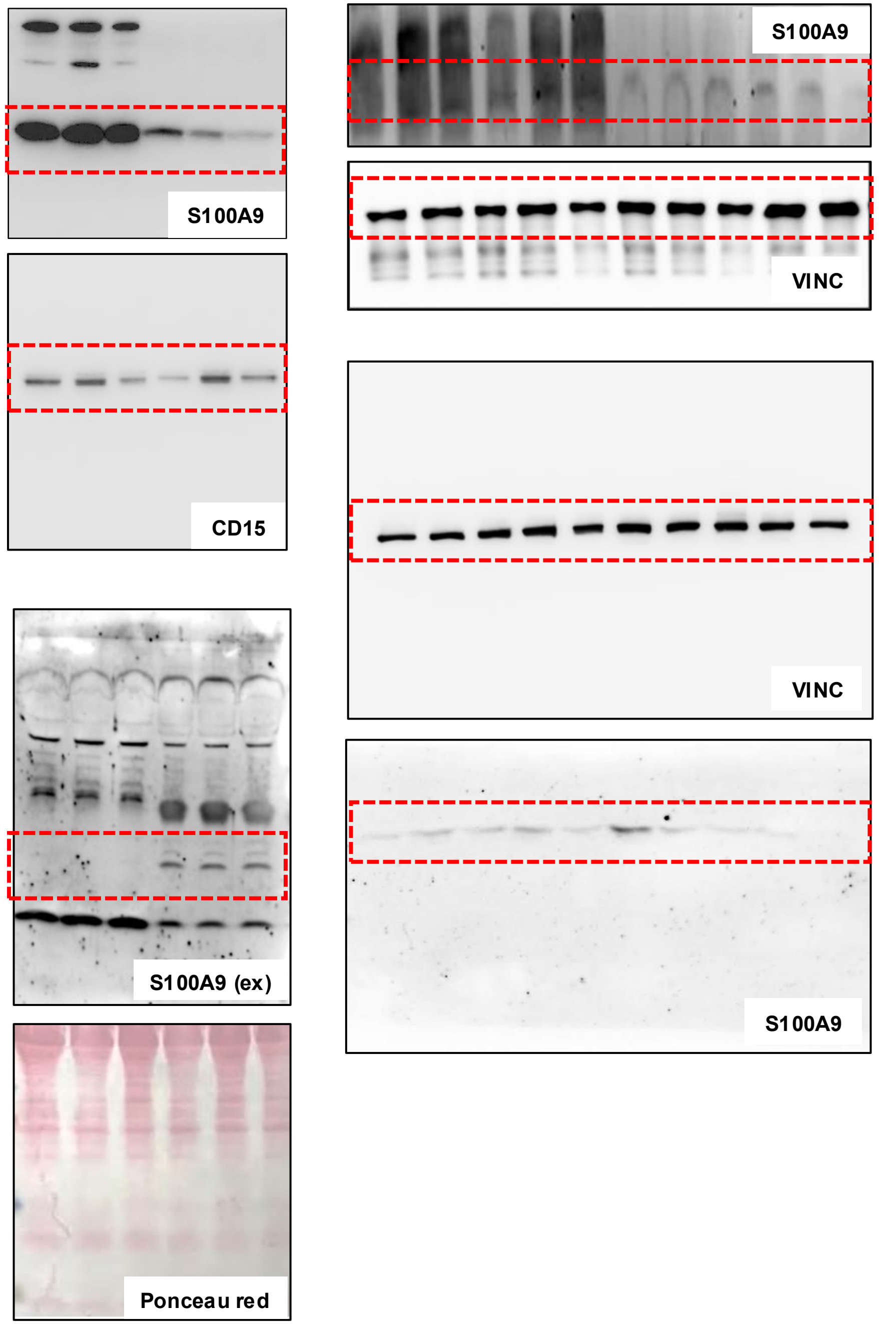

FIGURE 3
extended Data

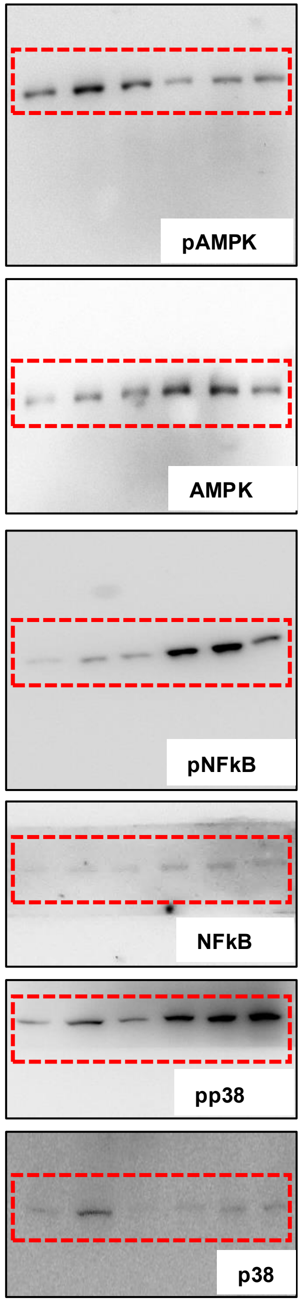

FIGURE 4
extended Data

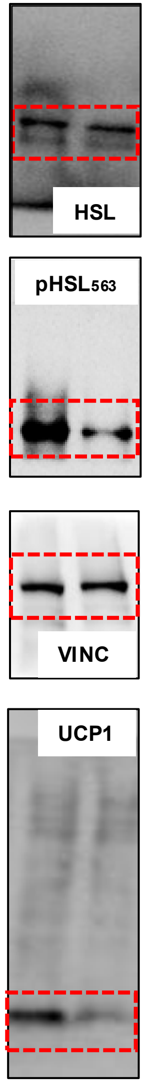

Supplement: Supplementary file 2 — Original Western Blots [file 41419_2026_8806_MOESM2_ESM.pdf]
